# Dichotomous sperm in Lepidopteran insects: a biorational target for pest management

**DOI:** 10.3389/finsc.2023.1198252

**Published:** 2023-08-23

**Authors:** Rakesh K. Seth, Priya Yadav, Stuart E. Reynolds

**Affiliations:** ^1^ Department of Zoology, University of Delhi, Delhi, India; ^2^ Department of Life Sciences, University of Bath, Bath, United Kingdom; ^3^ Milner Centre for Evolution, University of Bath, Bath, United Kingdom

**Keywords:** spermatozoa, sperm activation, sperm motility, initiatorin, serine endopeptidase, Lepidoptera, pest management, RNAi

## Abstract

Lepidoptera are unusual in possessing two distinct kinds of sperm, regular nucleated (eupyrene) sperm and anucleate (apyrene) sperm (‘parasperm’). Sperm of both types are transferred to the female and are required for male fertility. Apyrene sperm play ‘helper’ roles, assisting eupyrene sperm to gain access to unfertilized eggs and influencing the reproductive behavior of mated female moths. Sperm development and behavior are promising targets for environmentally safer, target-specific biorational control strategies in lepidopteran pest insects. Sperm dimorphism provides a wide window in which to manipulate sperm functionality and dynamics, thereby impairing the reproductive fitness of pest species. Opportunities to interfere with spermatozoa are available not only while sperm are still in the male (before copulation), but also in the female (after copulation, when sperm are still in the male-provided spermatophore, or during storage in the female’s spermatheca). Biomolecular technologies like RNAi, miRNAs and CRISPR-Cas9 are promising strategies to achieve lepidopteran pest control by targeting genes directly or indirectly involved in dichotomous sperm production, function, or persistence.

## 1 Lepidoptera as insect pests

Moths and butterflies, members of the insect order Lepidoptera, are among the most destructive pests of agricultural and horticultural crops. According to a recent report (1), the Cotton Bollworm, *Helicoverpa armigera*, is the most important single insect pest in terms of the number of recent publications, and three other species of lepidopteran larvae, the Diamondback moth (*Plutella xylostella*), the Tobacco Cutworm (*Spodoptera litura*), and the Fall Armyworm (*S. frugiperda*) are named among the ‘top ten’ list of all insect pests. *P. xylostella* alone is estimated to cause global economic damage of US$ 4-5 billion per year (2). Larvae of all four species attack multiple crops, undertake mass migratory movements, and build up invasive populations rapidly. *P. xylostella* is already a ubiquitous worldwide pest of field crops, while many other lepidopteran pest species, including all of those mentioned above, are currently undergoing range expansions (3, 4).

Unfortunately, existing control techniques cause significant environmental problems (5). Conventional chemical treatments may become less effective or even fail altogether due to the evolution of insecticide resistance (6) or because they disrupt populations of the pest’s natural enemies (7). Reliance on prophylactic applications of existing wide spectrum insecticides inevitably leads to further turns of the pesticide treadmill (8), exacerbating the risk of damage to non-target organisms and the natural environment (9). Ironically, anthropogenic declines of insect populations may affect natural enemies more than pests, thus worsening pest outbreaks (10).

It is clear that new approaches to the control of lepidopteran pest insects are urgently needed. While non-chemical control solutions aimed at the level of the agro-ecosystem are highly desirable (11), it is likely that technical interventions targeted specifically at the pest itself will continue to be required. Interference with reproduction has long been considered to have great potential for targeted pest control (12, 13). To avoid problems such as those mentioned above, control measures will need to be highly specific, yet easily tailored to particular pest species. They will also need to take account of the fact that unlike most other insects, in Lepidoptera it is the male that is the heterogametic sex, with most species in the order having a system of the ZW/ZZ or Z0/ZZ type (14).

In this paper we draw attention to the fact that all lepidopteran pests share unusual features of their male reproductive biology, especially their possession of two distinct types of spermatozoa, which we suggest constitutes an ‘Achilles heel’ that can be exploited to limit their fertility. With this in mind, we review the present state of knowledge of dichotomous spermatogenesis of Lepidoptera and the prospects for the targeted disruption of the genes that underlie it. Interference with male reproduction would be particularly useful in preventing the build-up of populations of migratory lepidopteran pests. This might be achieved by interfering with reproductive development in the targeted generation of insects using RNA interference or microRNAs, or by releasing genetically engineered insects that would suppress population growth, either by using second-generation Sterile Insect Technique (SIT) strategies, or homing gene drives, as will be discussed in Section 10.

## 2 Two types of sperm in Lepidoptera

**Figure 1:**
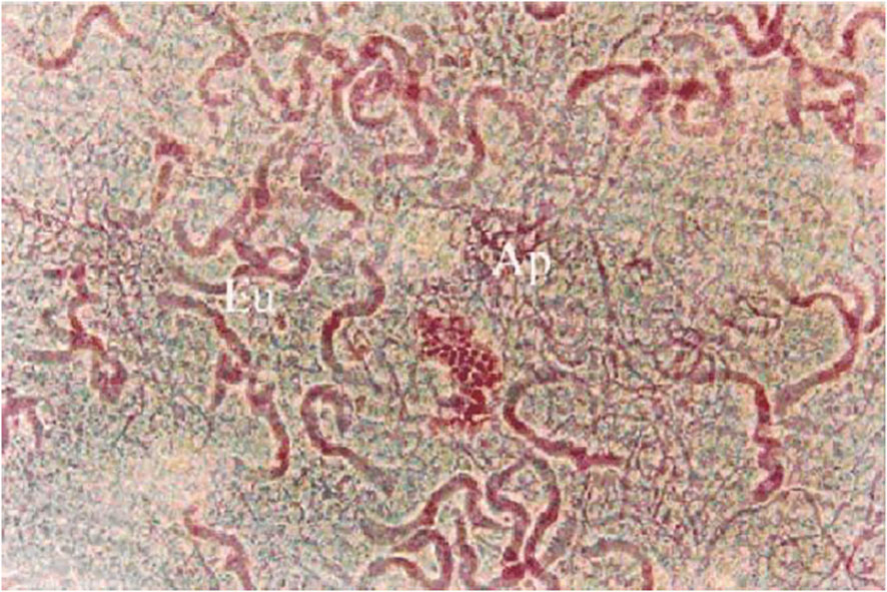
Eupyrene and apyrene sperm taken from the sperm storage organ (the duplex) of the male reproductive tract of *Spodoptera litura*. In this image, the apyrene spermatozoa (Ap) have already disaggregated and are visible as a tangle of many individual, thin profiles while eupyrene sperm (Eu) have not disaggregated and are seen as fewer thicker profiles, each representing a bundle of 256 co-oriented eupyrene sperm cells. Stained with lactic-acetic-orcein. From (15).

Moths and butterflies (Lepidoptera) differ from all other insects in that males produce two different sperm morphs (**Figure 1**), only one of which (eupyrene sperm or eusperm) is equipped with a nucleus, while the other type (apyrene sperm or parasperm) is completely anucleate (16, 17). Since only eupyrene sperm can fertilize eggs by contributing paternal genes, it must therefore be the case that apyrene sperm contribute to male fitness in other ways; these ‘helper’ functions are in fact essential, and successful fertilization of lepidopteran eggs requires both eupyrene and apyrene sperm to be transferred to the female (18, 19).

Sperm heteromorphism is not uncommon in the animal kingdom. It appears to have arisen independently on multiple occasions in a broad range of invertebrate taxa, as well as sporadically in several orders of insects (17). But lepidopteran sperm heteromorphy is extreme in character, and although highly variable, a surprisingly large fraction of lepidopteran sperm is made up of apyrene sperm. In a recent study of 17 species from 14 families, the apyrene:eupyrene ratio varied from 0.0264:1 (i.e. 2.6% apyrene) to 78.8:1 (98.7% apyrene) (20). In general, there is a steep increase in the proportion of apyrene sperm with increasing insect size, but even very small moths produce some apyrene sperm. Moreover, dichotomous sperm production is practically universal among Lepidoptera (21). The only known exceptions are a few members of a single genus in the most primitive and atypical lepidopteran family, Micropterigidae (22), and we may thus estimate that this kind of spermatogenesis arose 190-200 Mya, not long after the evolutionary origin of the order (23).

The selective forces that originally caused this evolutionary innovation are unknown, and the present-day adaptive function of apyrene sperm remains uncertain. Although it was suggested almost 40 years ago (24) that that the functional role of apyrene sperm is associated with post-coital sperm competition, solid evidence for this hypothesis is lacking (25). Numerous hypotheses for other possible ‘helper’ functions have been proposed. Of these, there is experimental support for only three; these are that apyrene sperm: (a) assist in transport or activation of eupyrene sperm, thus enhancing successful fertilization (18, 19); (b) enhance the fertilization success of eupyrene sperm by delaying female remating (26); and (c) facilitate dissociation of eupyrene sperm bundles through ‘stirring’ (27). Other plausible but as yet unproven hypothetical functions for apyrene sperm are listed in **Table 1**. It should be borne in mind that it is possible that different species of Lepidoptera make use of their apyrene sperm in different ways.

**Table 1:** Hypothetical adaptive roles of apyrene sperm in Lepidoptera.

Hypothetical adaptive roles of apyrene sperm in Lepidoptera	
*Supported by empirical evidence*	*Not supported by empirical evidence*
• assist in transport or activation of eupyrene sperm, thereby enhancing successful fertilization (18, 19)	• directly or indirectly capacitate (i.e. permit fertilization by) eupyrene sperm (28)
• delay female remating, thereby enhancing the fertilization success of eupyrene sperm (26)	• provide nutrient(s) for females and eupyrene sperm (29, 30)
• facilitate dissociation of eupyrene sperm bundles through ‘stirring’ (27)	• protect eupyrene sperm from being physically ejected by the female on remating (31)

Although they are still recognizable as ‘sperm’, apyrene sperm differ in many ways from eupyrene sperm. Their most obvious distinguishing characteristic is that they lack a nucleus and are markedly smaller in size than eupyrene sperm (32). Additionally, apyrene sperm do not possess an acrosome and their mitochondrial derivatives lack cristae. Importantly, eupyrene sperm, but not apyrene sperm, remain embedded in a substantial enclosing extracellular matrix when they are released from the testis into the male reproductive tract (33).

Apyrene sperm also differ from eupyrene sperm in that the two morphs express different proteins, thus potentially providing clues about their respective contributions to fitness. The proteomes of the two sperm types have been separately analyzed in two lepidopterans, the Monarch butterfly, *Danaus plexippus*, and the Tobacco Hawkmoth, *Manduca sexta* (34). Although only about 700 sperm proteins were identified in each case (almost certainly an underestimate of the actual total) we may for the moment regard the identified proteins as representative of the entire sperm-associated set. The eupyrene proteome is both more extensive (i.e. more proteins) and more complex (i.e. a wider range of predicted functions) than that of apyrene sperm, and only about half of the proteins found in eupyrene sperm are also present in apyrene sperm.

This implies the loss of expression in apyrene sperm of cellular proteins underlying functions that are no longer carried out by this sperm morph, reflecting an extensive functional streamlining during evolution of a eupyrene ‘ancestor’ present at the root of Lepidoptera. Presumably this absence of expressed proteins reduces the cost of producing those sperm that are useful only in a ‘helper’ capacity, but we may also speculate that it could be positively advantageous for the apyrene sperm *not* to display certain proteins found in eupyrene sperm, perhaps because they are the targets of inhibitory proteins expressed in the female tract as anti-sperm reagents during sexual conflict.

Although a set of proteins can be identified that is common to both types of lepidopteran spermatozoa (34), these are mostly those with predicted functions expected to be useful to any motile cell. Importantly, however, another subset of proteins is expressed only in apyrene cells; 61 proteins in *D. plexippus* are specifically enriched in apyrene sperm, while 146 proteins are specific to apyrene sperm in *M. sexta*. Approximately three quarters of the sperm proteins were functionally annotated according to the Gene Ontology (GO) classification system. This analysis showed that (as expected) apyrene sperm lack many proteins associated with the cell nucleus. Particularly interesting is the observation that several proteins with GO terms associated with nervous system and neuron development are upregulated in apyrene sperm compared with eupyrene sperm. The authors speculate that this may reflect the possibility that apyrene sperm may be specialized to “deliver neuro-endocrine active proteins that modulate female post-mating responses” (34).

## 3 Lepidopteran spermatogenesis

The underlying premise of the present review is that it may prove possible to interfere specifically with male reproduction in order to control lepidopteran pests, and that dichotomous spermatogenesis provides an unusually wide range of opportunities for such interference. To identify possible targets we will review the developmental biology of lepidopteran sperm formation.

**Figure 2:**
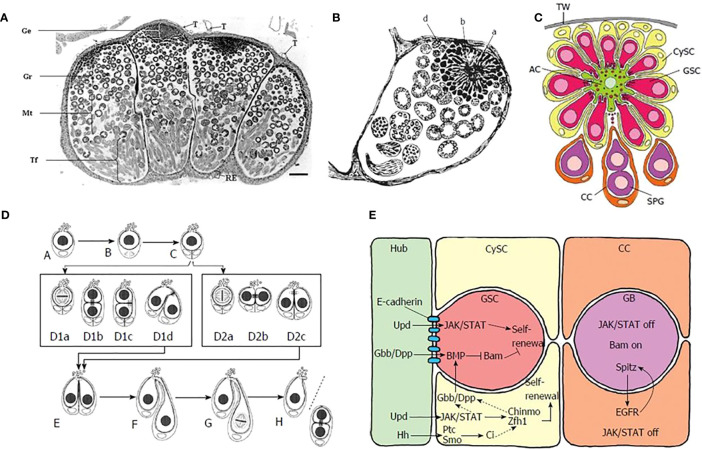
**(A)** Single unfused testis of *Bombyx mori*, light micrograph in cross-section. Note the four distinct lobes. [from Pereira et al. (2008), reproduced with permission]. **(B)** Verson’s (1889) drawing of a single lobe from *B*. *mori* testis, showing the apical complex **(A, B, D)**. The central apical cell **(A)** was considered by Verson to be a “germ cell” (“Keimzelle”) with radial extensions. What are now understood to be the true germline stem cells were then described as clumps of protoplasm with nuclei **(B, D)**. **(C)** Schematic diagram showing cell types in the apical complex in the lepidopteran *Lymantria dispar*. TW = testis wall, AC = apical cell, CySC = cyst stem cell, GSC = germ germline stem cell, CC = cyst cell, SPG = spermatogonia, [from Dorn and Dorn, 2015, reproduced with permission]. **(D)** Division of germline stem cells (GSC) in *L. dispar*. A-C: division of single cyst stem cell; D1a-D1d: asymmetric division of GSC; D2a-D2c: symmetric division of GSC; E-H self-renewal of GSC (on left side of cell pair) and formation of gonialblast (on right side of cell pair). [from Dorn and Dorn, 2015, reproduced with permission]. **(E)** Cell signaling within the apical complex: Hub, Apical cell; CySC, cyst stem cell; GSC, germline stem cell; CC, Cyst cell; GB, gonialblast. Color coding of cells is same as in **(C)**. [from (35), reproduced with permission].

The earlier stages of sperm cell differentiation in insects, in which male germ cells give rise to spermatogonia, are in general similar in Lepidoptera to those seen in other insects, although the spatial disposition of cell types in the lepidopteran testis is different (35). Both eupyrene and apyrene spermatozoa derive from the same bipotential primary spermatocytes and are produced in the same testicular follicle (there are four of these in each testis - **Figure 2A**), but the individual follicles each contain many individual testicular cysts (**Figure 2B**). These cysts are continuously produced from a late embryonic stage, being formed from a single apical complex in each follicle, in which a single large apical cell (variously called the Apical Cell (AC), Hub Cell or Verson Cell) is surrounded by numerous Germline Stem Cells (GSC), each of which is intimately associated to the AC through cellular projections, as well as with a single less closely-associated Cyst Stem Cell (CySC) (**Figure 2C**).

The GSC, custodians of the genome, and the CySC, which produce the cysts within which the germ cells are maintained in an undifferentiated, self-renewing state, reside within a privileged ‘stem-cell niche’. This is defined by soluble signals due to various secreted growth factors as well as contact signals due to cell surface proteins on the apical projections of the GSC that extend into the cell bodies of the AC. These projections undergo a process that resembles autotomy, in which cellular materials from the GSC are transferred to the AC and *vice versa*. Some information about the probable nature of both kinds of stem-cell-maintaining signals is shown in **Figure 2D**; much of this information is derived from genetic studies on *Drosophila melanogaster* (35–38) and although it is probable that the same signals are used in Lepidoptera, this is not yet certain.

Throughout life, GSC divide mitotically and asymmetrically, on each occasion producing one daughter cell that maintains the stem-cell state (this is the cell that maintains cellular contacts with the AC) and one cell that now becomes a gonialblast, which no longer maintains contact through cellular projections with the apical cell - **Figure 2E**. The gonialblast now progresses to a further mitosis that produces the first pair of spermatogonia, which then proceed through an additional five further rounds of cell division, eventually giving rise to a clone of 64 spermatogonia from which 256 spermatocytes are derived through the twin reduction divisions of meiosis (39).

**Figure 3:**
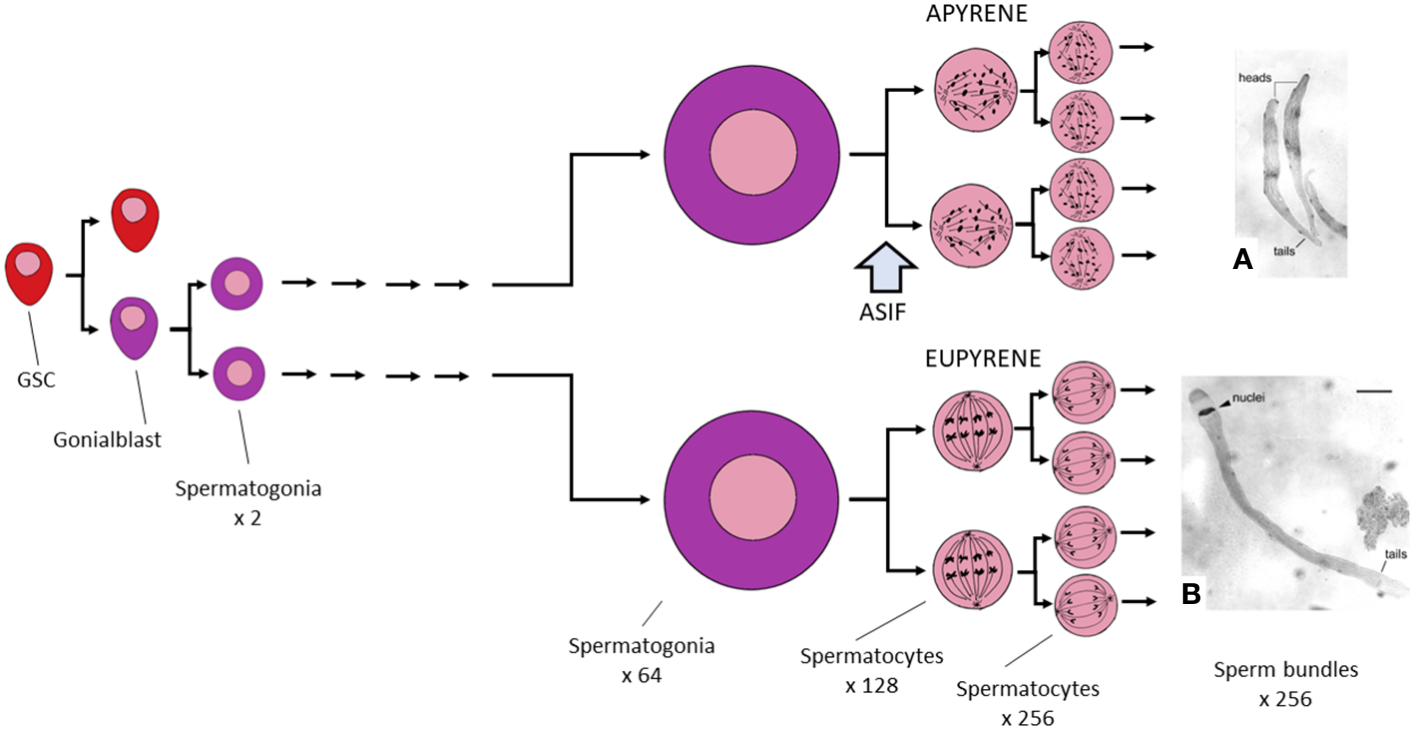
Graphic summary of Lepidopteran spermatogenesis. Germline stem cells (GSC) divide asymmetrically to produce a single self-renewing GSC and a single gonialblast. The latter divides to produce two spermatogonia, which then grow remarkably in size while also undertaking five rounds of mitosis. Development is indistinguishable in cysts that will eventually produce eupyrene and those that lead to apyrene sperm. Spermatogonia now enter the prophase of meiosis I, but arrest at this point until the block on meiosis is lifted, probably by exposure to ecdysteroid in the absence of juvenile hormone (JH). At this time, the spermatocyte will become committed to producing a eupyrene sperm unless it is also exposed to a (hypothetical) apyrene sperm stimulating factor (ASIF) that acts to commit the cell to producing an apyrene sperm (see the text for further discussion of this point). From this time onward, eupyrene and apyrene cysts differ in many ways. **(A)** shows two apyrene sperm bundles; **(B)** shows a single eupyrene sperm bundle, both from* Ephestia kühniella*; **(A, B)** are from reference (40), reproduced with permission.

Up to this point, the developmental progress of all testicular cysts is the same. But from here, eupyrene- and apyrene-destined cysts differ (**Figure 3**). While eupyrene meiotic divisions are regular in pattern, leading to the formation of pear-shaped spermatids, each of which contains a spherical nucleus, apyrene divisions exhibit an unusual distribution of chromosomes, which fail to pair properly, or perhaps at all (17). Although a spindle is present in the apyrene metaphase, it contains only polar microtubules, and the chromosomes appear to be unable to attach to them. As a result no conventional metaphase plate is formed, and the chromosomes segregate abnormally (41). The nuclear fragments that are reconstituted in the four resulting spermatids thus do not contain a full complement of chromosomes and they are later extruded from the cell during spermiogenesis, leaving the cell to develop as an anucleate but nevertheless recognizable, motile spermatozoon.

Initiation of spermatogenic development in Lepidoptera is a biphasic process; spermatogonia are first produced early during development, even before hatching, and are continually produced in the testes thereafter. Each gives rise to a clone of 64 spermatogonial cells, which begin to transform into primary spermatocytes. The cysts containing these clones now enter a state in which further development is blocked. The spermatogonial chromosomes undergo a limited condensation recognizable as the diffuse prophase of meiosis I, but then fail to condense further and the cell does not enter metaphase (42). The cessation of development at this stage requires the meiotic arrest gene *always early* (*aly*) (43).

**Figure 4:**
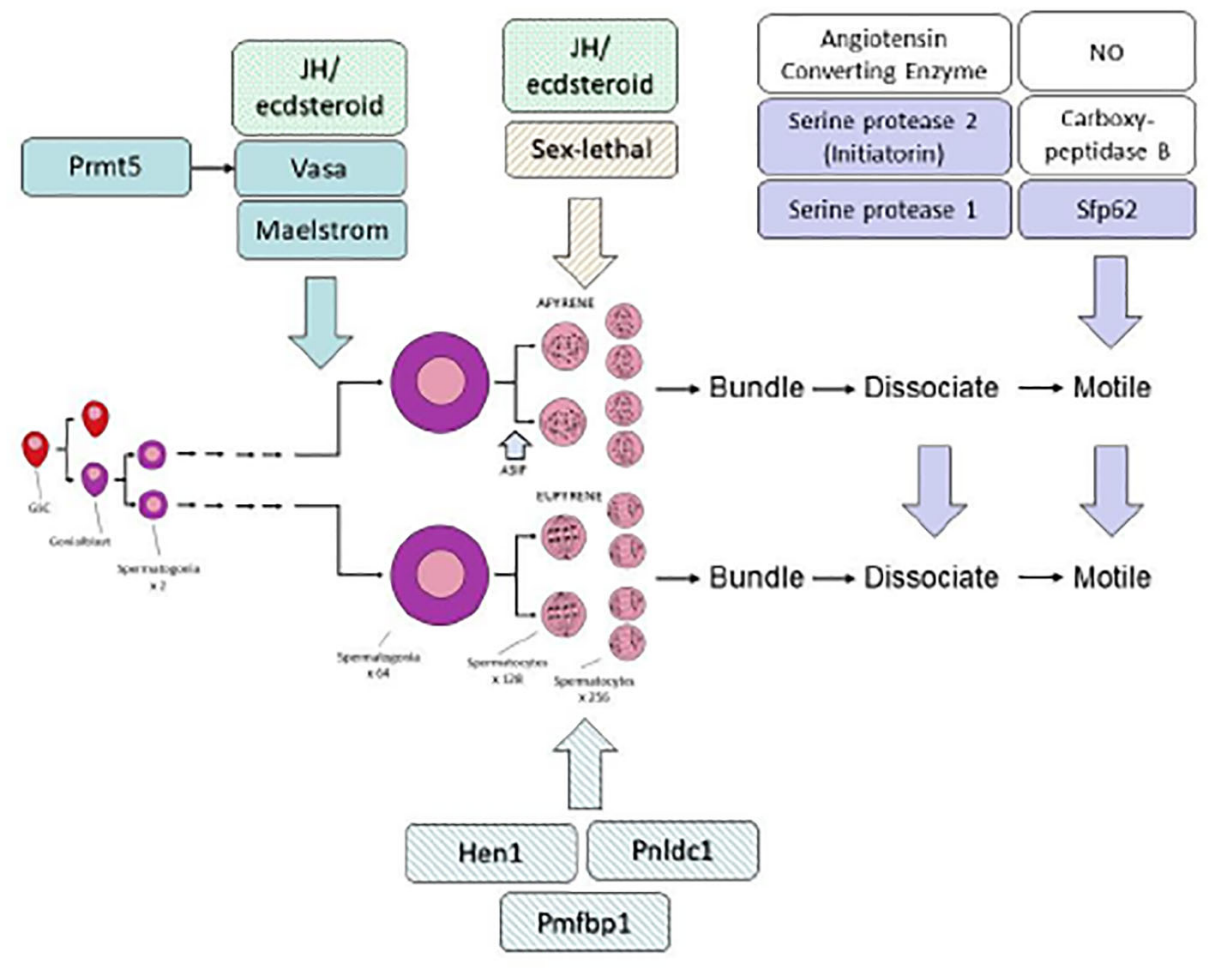
Hypothetical scheme to illustrate the regulation of sperm formation and activation. Endocrine control of spermatogenesis and spermiogenesis involves both ecdysteroid and JH as discussed in the text. Three gene products, Prmt5, Vasa and Maelstrom are required for normal formation of both eupyrene and apyrene sperm; three gene products, Hen1, Pnldc1 and Pmfbp1, are specifically required specifically for normal eupyrene sperm, and one gene product Sex-lethal, is required for normal apyrene sperm. Three gene products, Serine protease 1, Serine protease 2, and Sfp62 have been shown through gene editing to be required for normal sperm maturation (i.e. activation of motility and dissociation of sperm bundles). Further contributions to sperm maturation are due to additional enzymes and cellular signaling pathways as indicated by open boxes, but these have not yet been confirmed through gene targeting. In general, not enough is known to be able to indicate genetic control hierarchies with confidence, but Prmt5 is known to act through its action on Vasa. There is no doubt that the scheme is incomplete and that other genes will be found to affect the formation and maturation of lepidopteran sperm. For further details see text.

The arrested spermatocytes then persist in the same state (i.e. in prophase I) throughout larval life. There is good evidence from *M. sexta* that Juvenile Hormone (JH) is involved in blocking the further progress of sperm development in larvae, while it is the twin surges of the molting hormone 20-hydroxyecdysone (20HE) in the absence of JH that precede pupation, which appear to be responsible for the block’s removal (44). It is a reasonable hypothesis that the repression of spermatogenesis (like that of metamorphosis) is mediated by the JH-induced transcription factor Krüppel-homologue 1 (Kr-h1) (45), but this has not been shown. **Figure 4** shows a hypothetical scheme that attempts to integrate various aspects of current understanding of the regulation of male reproductive function in Lepidoptera.

JH/ecdysteroid control of sperm production at this larval checkpoint may be general among lepidopterans; a number of studies have indicated that resumption of spermatogenesis in the larvae of various species of Lepidoptera is dependent on ecdysteroid hormones and the absence of JH (e.g (46, 47). In some species, the requisite ecdysteroids are synthesized within the testis (48–52), although this is probably not the case in *M. sexta* (53). Moreover, although under experimental conditions (isolated abdomens) it appears that continued exposure to ecdysteroid is needed for induced eupyrene spermatogenesis to continue in *M. sexta* prepupae (44), once the massive prepupal ecdysteroid surge has occurred and pupation has taken place, no further exposure to either ecdysteroid or JH is required (53).

The cellular nature of the larval barrier to spermatogenic progress is still not known, but it is now generally accepted that the metamorphic developmental processes causing the transformation of the larva to the pupa are initiated when a surge of ecdysteroid in the absence of JH (the so-called ‘commitment peak’) results in a sharp decline of Kr-h1 expression. This in turn allows the transcription for the first time of the pupal specifier gene *broad* (54), a key component of the regulatory gene network that controls metamorphosis (55).

Still another possibility is that the resumption of eupyrene meiosis during the pre-pupal period requires not just ecdysteroid but also an additional signal mediated by the insulin/IGF signaling (IIS) pathway indicating the availability of adequate nutritional resources. In *Drosophila*, signaling by an Insulin-like Growth Factor (IGF) is required to maintain the proliferation of GSC in the testis, and also to permit the remarkable enlargement of spermatocytes prior to the re-initiation of meiosis (56). In *B. mori*, 20HE stimulates the production of Insulin-like Growth Factor-Like Peptide (IGFLP), which is synthesized in the fat body (57) and also in the testis wall (58). It is released in massive amounts during the first 5 days of pupal-adult development, when its concentration in male hemolymph reaches levels of >100nM (57). In male silkmoths, this growth factor acts in concert with 20HE to promote growth and development of the male genital disk to form the male reproductive tract (59). These actions of IGFLP are mediated by the IIS pathway, while those of 20HE are mediated by the Mitogen-Activated Protein Kinase (MAPK) pathway. It appears that IGFLP causes increased phosphorylation of the cell signaling transducer AKT, a serine/threonine protein kinase whose downstream growth and development actions are in turn mediated by the Target Of Rapamycin (TOR) and/or inactivation of the Forkhead-related FOXO family of transcription factors (59).

The commitment of spermatocytes to differentiate into eupyrene or apyrene sperm depends on the time at which the process is initiated. The first testicular cysts to resume spermatogenesis produce only eupyrene sperm. Later, the exact timing depending on the species, there is a developmental switch that results in the production of only apyrene cysts in the testis (60). In the Codling Moth, *Cydia pomonella*, eupyrene spermatogenesis begins in the last larval instar and ceases after pupation, while the later apyrene developmental process begins at about the time of pupation and continues to operate throughout the adult stage (61). Although there are differences in detailed timing, the switch from eupyrene to apyrene spermatogenesis occurs in much the same way in *B. mori* (62) and in *M. sexta* (53).

It was originally suggested more than 40 years ago (61) that a hypothetical circulating Apyrene Spermatogenesis Inducing Factor (ASIF) acts on primary spermatocytes causing them to become committed to develop into apyrene sperm cells. In *C. pomonella*, ASIF activity is first detected in hemolymph on the fourth day of the fifth (final) larval instar. Unfortunately the nature of ASIF remains unknown. The disappearance of JH from hemolymph is almost certainly involved. Evidence from the saturniid silkmoth, *Actias selene* (63), as well as *C. pomonella* (42, 46, 64), indicates that apyrene spermatogenesis occurs only in those insects that are committed to form a pupa, and we may suspect that the signals leading to apyreny are the same as those that initiate metamorphosis. If the absence of JH were all that mattered, then transplanting testes into organ culture without the hormone ought to be sufficient to initiate apyrene divisions, but this does not happen. In organ culture experiments using testes from *C. pomonella* (46) as well as *B. mori* (65), hemolymph is indispensable for proper spermatogenesis of either type, but addition of hemolymph that is predicted to contain ASIF is unable to change the developmental fate of cysts explanted into culture, and whether a cyst produces eupyrene or apyrene sperm depends only on the age and developmental progress of the donor of the cyst (66).

Once the eupyrene-apyrene switch has occurred, ecdysteroids and glucose are each able to increase apyrene sperm production in *in-vitro* cultivation of spermatocytes in *B. mori* (67), but this only occurs when cultured testicular tissue has been taken from testes in which apyrene development has already begun. As yet, we cannot say for certain that ASIF activity is not simply a property of a certain combination of known developmental hormones.

As noted above (section 3.2), the testis sheath of *B. mori* synthesizes an IGF-like peptide, IGFLP, the temporal pattern of expression of this peptide by the testis wall being limited to a period of time roughly corresponding to the initiation of apyrene sperm development; it has been tentatively suggested that IGFLP might be a paracrine regulator of apyrene meiosis (58). So far as we are aware, there is as yet no confirmation of this interesting hypothesis.

### 3.1 Spermiogenesis

The completion of meiosis in the primary spermatocyte results in the formation of four immature spermatozoa, or spermatids, which are initially round or pear-shaped. Spermiogenesis is the process whereby spermatids continue to develop, thereby acquiring the necessary apparatus of motility and fertility, especially a long, thin shape. It begins even before meiosis is completed and while the four sibling spermatids are still connected by cytoplasmic bridges. The transformation to the mature eupyrene spermatozoon involves a change in shape of the nucleus, production of an axonemal complex, fusion of mitochondria to form a nebenkern, formation of an acrosome, and the extrusion of cytoplasm by a process of ‘cytoplasmic squeezing’ that is accomplished by a network of actin filaments within the surrounding sheath cells. Apyrene spermiogenesis is similar, except that the final spermatozoa are shorter than in eupyrene sperm, and that the apyrene spermatids have multiple nuclear fragments instead of a single nucleus. These nuclear fragments are eliminated from the developing spermatid during cytoplasmic squeezing. All this has been previously reviewed (17).

While they remain in the testis, lepidopteran sperm cells of either type remain in their bundles of 256 spermatozoa. Neither kind of spermatozoa is observed to be motile within the cyst during the time that elapses between prepupa and adult eclosion. Neither eupyrene nor apyrene sperm are released from the cyst into the Upper Vas Deferens (UVD) of the male tract until morphological development of sperm is complete, and even then, they must await the receipt of an unknown developmental signal (19, 62). The nature of this signal is unknown, but its expression appears to be subject to a circadian rhythm (see below). On the other hand, mating does not appear to affect rate of release of sperm from the testis (15).

Apyrene bundles disaggregate in some species as they leave the cyst, the anucleate spermatozoa separating as they cross the basement membrane, or in other cases, during their passage down the UVD. By contrast, nucleated eupyrene spermatozoa do not separate as they are released from the follicle but remain confined in bundles both during storage and right up to the time of ejaculation into the spermatophore. They are held together by a substantial surrounding matrix of extracellular materials, which is enzymatically removed only when the bundles have been transferred to the female. The removal is effected by a digestion-like process that can be mimicked by immersing the sperm in a solution of the mammalian serine endoprotease trypsin (33, 68, 69). In *M. sexta*, eupyrene sperm only become separated into individual separate spermatozoa some hours after their transfer within the spermatophore into the female bursa (70).

Apyrene sperm begin to swim after being packaged in the spermatophore, soon after they have been transferred to the female’s bursa copulatrix. By contrast, eupyrene sperm remain immotile until much later, only beginning to move at the same time as they are being released from their bundles, the exact timing depending on the species, Even when they become motile, the swimming speed of eupyrene sperm is “slow” compared to that of apyrene sperm (68).

In those lepidopteran insects which undergo a developmental diapause, a developmental pause in spermatogenesis may occur that is completely different from the block that is seen in spermatocytes of normally developing larvae between the initiation of spermatogenesis in the embryo and its resumption in the last larval stage.

Some species, such as *M. sexta*, undertake an environmentally-determined pupal diapause, a state in which all external signs of metamorphic development cease, because the ecdysteroid titer fails to rise again after the larval-pupal molt. In the testis, however, stem cell divisions, the production of gonialblasts, and their further development towards spermatocytes, continue to occur throughout the diapause period, though neither eupyrene nor apyrene sperm are produced because in both cases the process is terminated before spermiogenesis can be completed. This is of some interest, because pupal diapause appears to extend exactly that developmental state in which both eupyrene and apyrene developmental pathways co-exist. In the case of eupyrene cysts, meiotic divisions have already been under way for some time by the time that pupal ecdysis occurs but would normally cease to be observed at around the time of pupal ecdysis. In diapausing males, eupyrene metaphases continue to be observed until pharate adult development is resumed, but all the resulting spermatids are subsequently aborted at an early stage of spermiogenesis, when they undergo a form of programmed cell death in which the developing sperm cells are destroyed and resorbed (53). In this case, therefore, continued initiation of eupyrene spermatogenesis evidently does not depend on the maintained presence of ecdysteroid, although it appears that survival of the developing spermatids does require the hormone. Development along the apyrene pathway also continues to occur, but in this case the resulting spermatocytes lyse before characteristically disrupted apyrene metaphases can be observed. This implies that their requirement for ecdysteroid (or something else that depends on ecdysteroid) differs from that of eupyrene spermatocytes. In most cases where the matter has been investigated in other Lepidoptera, pupal diapause is also due to lack of ecdysteroid and is accompanied by a similar pattern of continuing but disrupted spermatogenesis (e.g. *Agrius convolvuli* (71)). In *M. sexta*, 20-HE appears to provoke the re-initiation of spermatogenesis in diapausing testes by enhancing metabolism of glycogen in testicular sheath cells (60). This would not be surprising, particularly since we have noted above (see section 3.3) that nutritional signals *via* the energy supply-regulating IIS pathway appear to be required for earlier stages of spermatogenesis. It is also possible, however, that the change in glycogen deposits seen at this time is a consequence, rather than a cause of the changing developmental programme.

In the case of those lepidopterans that diapause in the last larval stage, such as in *C. pomonella* (46) or the Wax Moth, *Galleria mellonella* (72) it appears that diapause is the direct consequence of an elevated JH titer. This results in the suppression of the twin ecdysteroid peaks that trigger metamorphosis (73). Despite this difference, the effect on spermatogenesis is like what happens in pupal diapause; the developing spermatocytes of both eupyrene and apyrene type degenerate within the testicular follicles before undertaking meiosis, apparently due to the triggering of apoptotic programmed cell death (72, 74).

Diapause in the adult stage is different again. In the butterfly *Polygonia c-aureum*, adult diapause is the direct consequence of a lack of JH and is unaffected by ecdysteroids. Application of a JH analogue (methoprene) promoted sperm release from the testis into the male reproductive tract and also increased the mass of the simplex region of the tract (75).

## 4 Development of the male reproductive system

Bilaterally paired testes are separately recognizable in lepidopteran larvae from the earliest stages of development, but in many species (with the notable exception of *B. mori*) the two testes fuse to form a single testis as the larva transforms to a pupa (76, 77); this is presumably a metamorphic response to the ‘commitment peak’ of ecdysteroid in the absence of JH, followed by the larger surge of both hormones that follows during the prepupal period, but this has not been directly shown. The functional significance of testicular fusion is obscure. When testicular fusion was prevented surgically in *S. litura*, the total number of sperm bundles formed in the testes was reduced by about 60%, while those experiencing the same surgery but in which testis fusion still occurred did not experience any reduction and the apyrene:eupyrene ratio remained unchanged (78).

A transcriptomic study of testicular fusion in *S. litura* (79) has shown that fusion is accompanied by increased expression of a large number of genes, notably including those encoding cytoskeletal proteins (presumably involved in sperm movement) as well as genes involved in both the ecdysteroid and JH signaling pathways. The upregulation of several genes relating to chitin metabolism is unexplained but may relate to enhanced tracheation of the testis. Particularly interesting is that several genes related to Extracellular Matrix (ECM) proteins (collagens, laminins, proteoglycans and glycoproteins), receptor integrins and ECM-remodeling proteins including Matrix Metalloproteinases (MMP) were all upregulated during fusion. A broad-spectrum MMP inhibitor, GM6001, was found to prevent testis fusion, although it also caused many other pupal malformations.

Although the testes themselves develop early and grow proportionately throughout larval life, the rest of the lepidopteran male reproductive system is at this time present only in the form of reproductive *anlagen*, the male genital disks. As noted above, high circulating levels of IGFLP just after pupation in *B. mori* may have a role in the rapid growth and development of the male tract from the male genital disk during pupal-adult development. It has been shown that the titer of IGFLP in male silkmoths is maximal during early pupal life (days 1-5) (59), and concentrations of the peptide similar to those seen *in vivo* are able to promote the growth (measured as protein synthesis) of male genital disks *in vitro*. 20HE, also present in the testis at this time, separately promotes elongation of the genital disk, and acts co-operatively with IGFLP to further promote protein synthesis. The two hormones act via separate pathways; the actions of the IGF-like peptide are mediated by the IIS (insulin-IGF signaling) pathway, while those of 20HE occur via activation of the MAPK pathway. The effect on male fertility of targeted disruption of the IGFLP gene is discussed in section 9.

## 5 The sperm’s journey from testis to oviduct

In *S. litura*, around 3300 eupyrene bundles and 10,000 apyrene bundles are already present in the fused testis of the newly emerged adult male moth (80). Once they are mature, both kinds of sperm migrate into the UVD. Eupyrene sperm are first to leave the testis, and they are followed after an interval of several days by apyrene sperm. The release of both kinds of spermatozoa from the testis appears to be negatively regulated by ecdysteroid hormones; like adult eclosion (81), sperm release does not occur until the circulating ecdysteroid titer begins to fall prior to adult emergence and is delayed by exogenous 20HE (82).

On leaving the testis, eupyrene sperm remain within their original bundles in the male reproductive tract, while apyrene sperm dissociate either as they leave the testis or shortly afterwards. The process by which by both apyrene and eupyrene sperm enter the UVD involves intact sperm bundles crossing the cyst’s terminal epithelium, a cellular barrier without a basement membrane on either side, which separates the interior of the testicular cyst within which the sperm have developed and the lumen of the UVD. The sperm bundles appear to pass between the epithelial cells. The motive force for this movement appears to come from ‘head cyst’ cells that force the sperm bundle forward into the epithelium from the end of the bundle that is furthest from it; this involves the head cyst cell’s actin cytoskeleton and is inhibited by cytochalasin (83). RNAi-mediated reduction of β-actin levels in these cells specifically prevents sperm release (84). It is uncertain whether undulating sperm motility is involved in their leaving the cyst. According to (85), as they are escaping from the cyst in which they were formed, apyrene (but not eupyrene) sperm of *B. mori* are briefly motile, and this may assist them in entering the UVD. Subsequently, however, as they descend within the male tract, they lose this power of motion and revert to a condition of immobility.

In diverse Lepidoptera, the process of release of sperm from the testis described above is subject to a daily rhythm (e.g. *Ephestia kühniella* (86),; *Lymantria dispar* (87),; *C. pomonella* (88),; *S. littoralis* (89),; *S. litura* (15), and *Polygonia c-aurum* (90),. This has been shown in the case of *L. dispar* to be directed by an endogenous circadian rhythm located in the cells of the male reproductive tract (91). If the moths are kept in constant light sperm release is adversely affected and the male moths are effectively sterilized.

Once they have entered the UVD, both types of sperm move towards the sperm storage organ (i.e. the duplex or the seminal vesicle [SV] according to species) and continue their development towards the fully mature state as they go. In *S. litura*, the released sperm are stored within the duplex of the male tract until they are ejaculated during mating (15), whereas in *M. sexta* and *B. mori* they are stored in the SV. Their movement along the tract is not self-powered because at this stage neither apyrene nor eupyrene sperm are motile; instead, the vas deferens itself undertakes rhythmic myogenic contractions that propel sperm along its lumen; these myogenic contractions are themselves subject to an endogenous circadian rhythm (92). While in the UVD, sperm are subjected to acidic conditions due to proton pumping into the lumen by a vacuolar ATPase (V-ATPase) located in the cells of the UVD wall; this too is subject to a daily rhythm (93, 94). The sperm are also exposed during this journey to various seminal fluid proteins (SFPs) including (perhaps surprisingly) yolk proteins, which are secreted by the cells of the male tract wall, this secretion also being subject to a daily rhythm (95).

During mating, seminal secretions are transferred from the male into the female reproductive tract and coagulate there to form a solid structure, the spermatophore, which is located within the bursa copulatrix. The process by which this happens in *M. sexta* has been described in detail (70). The spermatophore is a highly organized structure consisting of an outer wall which is further divided into several layers and two internal matrices that contain sperm and seminal fluids (96, 97). Sperm enter the spermatophore in the later stages of its formation (70), and it is here that they become motile. The contents of the inner matrices become reduced in volume at the same time as the sperm become active. In *M. sexta*, ejaculated apyrene sperm quickly acquire the power of movement, but eupyrene sperm become motile more slowly over several hours. The greater motility of the apyrene sperm has been suggested to facilitate the dissolution of the eupyrene sperm bundles at this time (68). There is a soft plug at the end of the spermatophore from which it was filled and through which the sperm will exit to enter the female seminal duct. Spermatophorins, the structural proteins of spermatophores, are derived from secretory products of the male accessory glands. A secretory protein of 23kDa rich in glutamine (15.4%) and proline (25.2%) is found in secretory vesicles of only one cell type in the male accessory glands, and once secreted is restricted to a discrete layer in the spermatophore outer wall (98). The spermatophore contains granules of glycogen that presumably supply metabolic substrates to sperm (96, 97).

In *S. litura*, about 48% of eupyrene sperm bundles have been dissociated within the spermatophore by 15 min after the completion of mating (ca. 82% of eupyrene sperm bundles by 30 min). Eupyrene sperm bundles disappear from spermatophore within 6h post copulation (80). Apyrene spermatozoa begin to leave the spermatophore soon after termination of copulation. By 30 min, many apyrene sperm have already left the spermatheca. Eupyrene sperm, by now released from their bundles, follow shortly afterwards. At 30 min after mating only apyrene sperm have reached the spermatheca, while at 45 min eupyrene sperm are also observed there. Most of both the apyrene and eupyrene spermatozoa reach the spermatheca within 12 hours after the end of copulation (80). As would be expected, these migratory processes occur more slowly in the much larger *M. sexta*, with apyrene sperm first entering the spermatheca at about 4 h after mating, while eupyrene sperm arrive there at about 11 h after mating (70).

Sperm of both kinds become less motile with increasing time after the completion of mating. In *S. litura*, 12 hours after copulation, 23.4% of eupyrene sperm were motile, while 82.7% of apyrene sperm were motile (80). With further increase in time, there was no significant change in the motility of eupyrene sperm but the motility of apyrene sperm decreased significantly. It is unknown why this decrease in motility occurs: possible hypotheses are that local stores of energy-yielding substrates are exhausted; that continued motility of spermatozoa is harmful; that the survival time of spermatozoa is programmed to be short; and that the female tract contains substances that are harmful to sperm. Some female moths do not survive long after egg laying, but others may live longer and undertake a further round of reproduction; in the Chinese Windmill, *Byasa alcinous*, a papilionid butterfly, no motile apyrene sperm from an initial single mating remained in the spermatheca 8 days after copulation (99). In some species, female moths discard surplus sperm from the spermatheca (31, 100).

## 6 Sperm activation in Lepidoptera: peptidases

Seminal Fluid Proteins (SFPs) play an important role in determining competitive fertilization success in many animals, especially those practicing internal fertilization (Simmons and Fitzpatrick, 2012). It has long been known that this is also true for Lepidoptera; it is now more than 80 years since Omura (101, 102) showed that when used in artificial insemination procedures, sperm taken directly from the male tract of *B. mori* were unable to fertilize eggs, but the addition of even a trace of prostatic secretions rendered them capable. It was anecdotally reported that surgical removal before mating of the paired male accessory glands of the Cabbage Looper, *Trichoplusia ni*, did not prevent successful transfer of sperm but resulted in their failure to migrate normally within the female tract (103).

Ascribed functions of SFPs encompass effects on both male and female reproductive systems. These include promoting the survival and viability of sperm within the male reproductive tract especially within sperm storage organs; modifying the structure of the outer surfaces on the spermatozoa of the individual male itself, (possibly in addition to those of other males) so as to influence sperm motility and capacity to undertake fertilization; as well as modulating the biosynthetic and physiological activities of cells in the walls of the female reproductive tract so as to promote female fertility through increased ovulation etc; protecting both the male and the female reproductive tracts against microbial infection; modulating female behavioral receptivity to remating as well as forming mating plugs that physically prevent remating; as well as affecting the longevity, feeding behavior and locomotory activity of female insects (103, 104).

The techniques of transcriptomics and proteomics have enabled rapid progress to be made in recent years in understanding both the nature of SFPs and also their significance for reproductive behavior and physiology. Most information is available for *Drosophila* (105) but other insect orders including Lepidoptera have also been studied (104).

There is good evidence that sperm activation in *Bombyx mori* is initiated by a trypsin-like protease first discovered by Omura (101), named initiatorin by Osanai (69), partially sequenced by Aigaki et al. (106), and fully characterized (as *Bombyx mori* male reproductive organ serine protease 2) by Nagaoka et al. (107). Proteins with similar amino acid sequences are found in other Lepidoptera, and CRISPR/Cas9 targeted mutation of the gene in both *B. mori* and *P. xylostella* resulted in male sterility (108). Initiatorin/serine protease 2 is a protease of the serine endopeptidase type, resembling trypsin and endopeptidase Arg-C, but differing from those enzymes by its strong preference for Arg–Arg sites in the substrate. Initiatorin is produced in the posterior part of the glandula prostatica and ampulla of the male tract and causes both apyrene sperm activation and eupyrene sperm bundle dissociation. It is synthesized as a protein consisting of three domains. The nascent pre-pro-protease undergoes scission of a putative signal domain (DI) to produce an inactive pro-protease form (DII-DIII) of 32 kDa which is stored in the glandula prostatica. Once secreted, the C-terminal of the DIII domain is cleaved by autolysis, causing activation of the enzyme (107). Activation takes place mainly in the spermatophore, yielding mature active proteins of 29 & 26 kDa, both of which are able to activate sperm.

*In vivo*, other factors are required in addition to initiatorin/serine protease2, and the activation of both apyrene and eupyrene sperm appears to be a multi-stage process. In particular, there is evidence for the involvement of other proteases. One of these is peptidyl dipeptidase A, commonly known as Angiotensin Converting Enzyme (ACE). This enzyme occurs widely among mammals in two forms, a double-domain somatic form (sACE) and a single-domain ‘testicular’ form (tACE) which is exclusively found in sperm, where it plays an essential role in fertility (109). In insects, ACE has been characterized from *Drosophila* (110) and shown to occur in the male reproductive system of several insects including lepidopterans (111). That ACE is transferred to the female during mating was shown for the Tomato Moth, *Lacanobia oleracea (* 112 *)*,; it was found that the enzyme’s activity increases in the bursa copulatrix during copulation, while it simultaneously decreases in the SV of mated males. In *B mori*, ACE exists in two isoforms; a double domain somatic form (sACE; 180 kDa) and a single domain male reproductive tract form (tACE; 110 kDa), both isoforms being transcribed from the same gene. Transcripts are found mostly in the seminal vesicle and ampulla. It has been hypothesized that ACE might play a role in sperm activation in *B. mori* (113). A specific ACE inhibitor, captopril, completely blocks the ability of extracts of the seminal vesicle (and also of trypsin) to activate apyrene sperm and to dissociate eupyrene sperm bundles. Because ACE is present in the seminal fluid that is used to test the sperm-activating ability of initiatorin (and trypsin), it is not possible to exclude the possibility that ACE might not act directly on sperm but instead acts cooperatively with initiatorin, or indeed might activate the latter.

Another proteolytic enzyme involved in sperm activation in *B. mori* is the M14 domain zinc metalloprotease, carboxypeptidase B (CPB) (114). CPBs with sequence similarity have been shown to be present in many lepidopteran species. In *B mori*, CPB is synthesized and stored in the SV and the ampulla, from which it is depleted during mating. The stored form is a 48kDa inactive proenzyme, which is specifically cleaved by initiatorin/serine protease 2 at the conserved CPB dibasic site Arg^109^-Arg^110^ to yield a highly active 32kDa product. At moderate concentrations, bovine trypsin also activates *B. mori* CPB, but the exogenous enzyme is less specific in its cleavage pattern and at high concentrations (unlike initiatorin) trypsin abolishes CPB activity.

## 7 Sperm activation in Lepidoptera: intracellular signaling

There is evidence that lepidopteran sperm activation may also involve cyclic nucleotides and other small molecule cell signaling pathways (115). Washed sperm are not activated by trypsin or prostatic extracts unless exogenous cyclic adenosine monophosphate (cAMP) is added, but cAMP alone is ineffective without the peptidase (69). It has been suggested that in *B. mori*, initiatorin/serine protease 2 acts, at least in part, by solubilizing glycoproteins on the sperm surface, thus revealing 60Å pores in the flagellar membrane, allowing the influx from outside of cAMP (85). cAMP signaling mediated by Protein Kinase A (PKA) and intracellular Ca^2+^ ion mobilization are known to be involved in the capacitation of mammalian (including human) sperm, but it should be emphasized that mammalian sperm capacitation is a complex and as yet imperfectly understood process (116, 117). Cyclic guanosine monophosphate (cGMP) also plays a role in activating mammalian sperm (118), but we do not know whether this signaling pathway operates in lepidopteran sperm.

Moreover, there is no available information on the cellular control pathways that would respond to either cAMP or cGMP in lepidopteran sperm. The tiny available quantities of semen that are available for analysis mean that it is difficult to measure the levels of intracellular second messengers or the activity of cellular signaling enzymes like PKA. It is possible that transcriptomics may yield useful information. Although it was previously thought that once meiosis has begun, developing sperm cells do not synthesize new mRNAs (119), it is now evident that at least some genes are newly transcribed during spermiogenesis (120). It must be borne in mind, however, that the relevant gene products for sperm activation may actually be manufactured in supporting cells rather than in the sperm cells themselves. Analysis of sperm proteins may prove to be a valuable technique. There is for example proteomic evidence that a cyclic nucleotide Phosphodiesterase (Pde1c) protein is present in *Drosophila* sperm (105). Pharmacological interference with intracellular signaling pathways may also be a promising approach to improve understanding of the activation and capacitation of insect sperm.

Calcium ion influx into sperm cells plays a key regulatory role in motility of sperm in mammals (121). As far as we are aware, there is no available information on the role of Ca²^+^ ions in lepidopteran sperm activation, but in the mosquito *Culex pipiens*, exogenous trypsin was found to activate a Protease Activated Receptor-2 (PAR2) like protein in order to confer motility on sperm, this being mediated by a signal transduction pathway involving Ca²^+^ influx and the activation of the intracellular signaling protein kinase enzyme MAPK, which in turn activates sperm motility (122). Little is known about this in lepidopteran sperm; the apyrene sperm of *M. sexta* become motile in the presence of the divalent cation ionophore A23187, but it is uncertain whether Ca^2+^ ions are responsible for activation (123).

Nitric oxide (NO) also appears to play a role in the acquisition of sperm motility in *Bombyx* and perhaps also in other Lepidoptera, but probably acts in concert with other activators (124). Unusually, in the male reproductive system of *B. mori*, Nitric oxide synthase (NOS) is an extracellular enzyme, though the supposed action of NO on sperm is intracellular. NO is present in seminal fluid, being synthesized in the glandula lacteola of the male tract and transferred to the female during mating. NO is a short-lived molecule in biological systems and therefore difficult to measure, but its presence can be inferred by measuring the levels of two stable metabolites, NO_2_^-^ and NO_3_^-^. These are present in the spermatophore, and also in the bursa after completion of mating, indicating the synthesis of NO in these locations during mating. The concentrations of NO_2_^-^ and NO_3_^-^ peak at the time of maximal apyrene sperm motility. Activation of apyrene sperm by Initiatorin or trypsin is delayed by NOS inhibitors such as N-omega-nitro-L-arginine methyl ester (L-NAME) treatment with carboxy-PTIO, a NO scavenger that transforms NO to NO_2_ without interfering with NOS activity, also inhibits sperm motility. On the other hand, application of NO donors (SNAP & NOC7) to immotile sperm did not on their own induce apyrene sperm motility, and prior treatment of apyrene sperm with a glandula prostatica homogenate preincubated with NO donors did not significantly change sperm motility.

On the basis of the above evidence, it has been concluded (124) that in *B. mori*, sperm NO is synthesized from arginine by NOS in the extracellular medium and enters apyrene sperm via the membrane channels revealed by Initiatorin action. Once within the sperm cell, NO acts as a regulator of cellular activities; its cellular target is not known. In mammalian cells, NO has many cellular actions (125), but by analogy with the case in which NO regulates human sperm motility, it is likely that in Lepidoptera modulation of protein phosphorylation within the sperm cell is involved (126). One possible target would be the production of ATP by the citric acid cycle within the sperm mitochondrial derivative. NO is known to regulate metabolism in this way in mammalian cells (127). Moreover, the NO regulatory system interacts with the proteolytic enzyme Initiatorin in another way; the enzyme acts to degrade extracellular proteins either present in the seminal fluid or in the walls of the spermatophore to release free arginine. This not only acts as substrate for NOS but also supplies sperm with the energy necessary for motility when it is further converted to 2- oxoglutarate by which feeds into the Krebs cycle (68, 128, 129).

## 8 Genes that control dichotomous spermatogenesis

Until relatively recently, knowledge of genes underlying male reproduction in insects was derived solely from only a few intensively studied model insects, especially *Drosophila melanogaster*. Although something was known of the genetics of the silkworm, *Bombyx mori*, this knowledge was limited and almost nothing was known about the genomes of pest species. Understanding accelerated remarkably when it became possible to use DNA hybridization methods to discover homologues of *Drosophila* genes in other insects. An example of this, relevant to lepidopteran spermatogenesis, was the identification of the *doublesex* gene (*dsx*) in the silkworm *Bombyx mori* (130). This autosomal gene is a key component of a conserved Gene Regulatory Network (GRN) that controls sex determination and is conserved in a wide variety of insects (131). In *B. mori*, the *dsx* gene is transcribed into alternative sex-specific mRNA isoforms, one of which, *dsxm*, encodes a zinc-finger DNA-binding protein that is responsible for the masculinization of those individuals destined to develop as males (132).

As we will see below, targeted disruption of *dsx* and other members of the sex-determining GRN can be used to produce sterile male insects. Unfortunately, however, interference with sex determination also interferes with genital anatomy and mating behavior, so that the resulting insects, although sterile, do not compete well for mates. It will therefore probably be better, where it is desired to interfere with pest fitness through SIT or homing gene drives, to concentrate on disrupting genes that operate specifically on sperm function, and which do not interfere with mating competence. Thus, in recent years much attention has been paid to the identification and disruption of such genes. This has been facilitated by a number of methodological advances, in particular RNA interference and CRISPR/Cas9 gene editing, which have allowed remarkable advances in understanding the genetics of various aspects of male reproduction in Lepidoptera, directly revealing what happens when particular genes are not expressed. Although some of these genes were initially identified by genetic screening in *Drosophila*, several were discovered as a result of studying the physiology and biochemistry of male reproduction.

### 8.1 RNA interference experiments

Probably originating as a natural defense against viruses, eukaryotes have developed the ability to recognize and destroy exogenous double-stranded RNA (dsRNA) using special dsRNA-recognition proteins and RNases; this involves the initial conversion of the dsRNA into short interfering RNAs (siRNA). Taking advantage of this, the RNA interference (RNAi) technique uses specifically designed exogenous dsRNAs to provoke the destruction of the target organism’s own single-stranded mRNA molecules, thus ‘knocking down’ the expression of the targeted gene (133, 134). RNAi studies have provided important information on genes that are important in lepidopteran spermatogenesis and sperm maturation. They are listed in the upper panel of **Table 2**. The list is disappointingly short. By comparison, RNAi studies on male reproductive function in non-lepidopteran insects have been more successful (lower panel of **Table 2**). The only promising lepidopteran study to date is one in which a dsRNA targeted against the gene *serine protease 2* was expressed in a RNAi-transgenic line of the Fall Webworm, *Hyphantria cunea*. This lack of success in knocking down the expression of male reproductive function through RNAi reflects the general finding that although particular species and genes vary in their response, lepidopteran pests are especially difficult to attack using RNAi (146). There are many possible reasons for this (147, 148), and the problem is discussed further below in Section 10.

**Table 2:** Targeting sperm-related genes using RNAi and miRNAs.

Species	Order, Family	Method	Mode of delivery	Gene Name	Gene Product	Function	Treatment effect	Effect on fertility	Reference
Lepidoptera									
*Spodoptera littoralis*	Lepidoptera, Noctuidae	RNAi	dsRNA *in vitro*	β*-actin*	β*-actin*	Cytoskeletal protein	inhibited release of sperm from testis into male reproductive tract (RT)	Not reported	(84)
*Spodoptera littoralis*	Lepidoptera, Noctuidae	RNAi	dsRNA *in vitro*	*period*	Period	Circadian timing	Delayed release of sperm from testis into male RT; delayed association of sperm with yolk protein; delayed rhythm of V-ATPase activity in male RT	Not reported	(93, 95, 135)
*Sitotroga cerealella*	Lepidoptera, Gelechiidae	RNAi	dsRNA by injection	*triacylglycerol lipase*	Triacylglycerol Lipase	lipolytic enzyme	impaired production and transfer of apyrene sperm	Reduced male fertility	(136)
*Hyphantria cunea*	Lepidoptera, Arctiidae	RNAi	transgenic *piggyBac* RNAi line	*serine protease 2*	Serine Protease 2	Accessory gland serine endopeptidase, activates motility in eupyrene and apyrene sperm	sperm are infertile, but the reason for this was not determined	Transgenic line shows dominant male infertility	(137)
Other insects									
*Drosophila melanogaster*	Diptera, Drosophilidae	RNAi	genetic RNAi line	Acp36DE	Acp36DE	glycoprotein transferred from male to female	Reduced sperm storage in female	Sperm are less competitive	(138)
*Anopheles gambiae*	Diptera, Culicidae	RNAi	dsRNA by injection	*zero population growth (zpg)*,	ZPG	gap junction protein, maintenance of GSC	No mature sperm produced	Males (M) infertile	(139)
*Athalia rosae*	Hymenoptera, Tenthredinidae	RNAi	dsRNA by injection	*boule (bol)*	Member of Deleted in Azoospermia (DAZ) gene family	Essential for meiosis	No mature sperm produced	No female (F) offspring, M offspring normal	(140)
*Diabrotica virgifera*	Coleoptera, Chrysomelidae	RNAi	dsRNA in food and also expressed by plants	*boule (bol)*	Member of Deleted in Azoospermia (DAZ) gene family	Essential for meiosis	Not reported	Reduced M fertility	(141)
*Aedes aegypti*	Diptera, Culicidae	RNAi	dsRNA by injection	*AMT1*	Ammonium transporter	cellular pH regulation	fewer sperm	Reduced M fertility	(142)
*Bactrocera dorsalis*	Diptera, Tephritidae	RNAi	dsRNA in food	*mitoferrin (mtf)*	Mitoferrin	iron transport	fewer sperm, more dead sperm	Reduced M fertility	(143)
*Bactrocera dorsalis*	Diptera, Tephritidae	RNAi	dsRNA by injection	*corazonin*	Corazonin	neuropeptide	lengthened mating duration in males	Not reported	(144)
*Frankliniella occidentalis*	Thysanoptera, Thripidae	RNAi	dsRNA delivered on nanoparticles	*fibre sheath CABYR-binding protein*	FSCB	sperm capacitation?	sperm fertility reduced	M-biased offspring	(145)

Gene expression can also be selectively inhibited through the RNAi-like action of microRNAs. These are endogenous small, non-coding RNAs (average size 22 nucleotides), which modulate gene expression (usually negatively) by interacting with mRNA transcripts of developmentally regulated genes. First described among insects in *Drosophila melanogaster* (149), miRNAs have since been found in all insects so far investigated, including Lepidoptera, in a limited number of conserved sequences. In *B. mori*, 118 conserved miRNAs and 151 novel miRNA candidates have been identified from the *B. mori* genome (150). miRNAs are produced through the action of the RNase III enzyme Dicer1, and this enzyme is essential for normal development in a number of insects including *B. mori* (151). It would be fair to say, however, that no coherent picture has yet emerged as to how their regulatory roles map onto the list of known insect miRNA sequences, and in some cases their target genes appear to differ even between closely related insect species.

It has been proposed (152) that miRNAs might be utilised as insect control agents, citing 28 studies of insect miRNA function in 12 species from 5 insect orders. The prospective value of this approach for Lepidoptera has been demonstrated (153) in a study that supplied the miRNA miR-2002b to larvae of *H. armigera* in artificial diet at sub-micromolar concentrations; this adversely affected both larval growth and development and also adult fecundity. A trypsin-like serine proteinase was identified as one of the miRNA’s targets. With relevance to the present paper, some miRNAs are known to regulate gene expression in male reproduction; although no lepidopteran example has yet been identified, a known case is the dipteran pest *Bactrocera dorsalis*, where miR-8-3p is known to be expressed in the testes and to downregulate expression of the Mitoferrin protein during spermatogenesis, adversely affecting the number and viability of sperm and thus reducing male fertility (143). Also in *B. dorsalis*, expression of the translational regulator *orb-2*, required for spermatocyte meiosis and subsequent spermiogenesis, is subject to the combined influence of two miRNAs, miR-125-3p and miR-276b-3p. Treatment of developing insects with the corresponding agomirs (chemically modified dsRNAs with cognate sequences, designed to extend their effective lifetime) reduces male fertility (154).

### 8.2 Gene editing experiments

In classical genetics, the genes that underlie specific traits are identified by randomly generating mutants and located within the genome by analysis of crossing experiments. The resulting mutant lines can also be used to unravel gene regulation and interactions. But this requires the maintenance of large numbers of genetic stocks of the species in question, something that is only possible for a few model organisms. Lepidoptera do not figure prominently in such work. Identifying the genes responsible for particular traits in non-model lepidopteran species thus requires a technique to target mutations to candidate DNA sequences.

Several methods for this were devised in the decade 2000-2010, e.g. using specially designed Zn Finger (ZNF) DNA-binding proteins (155) and customized TAL effectors. This is the TALENS system (156), which was successfully used to disrupt the gene encoding the *sex-lethal* (*sxl*) gene in *B. mori* (19). This sex-specific transcription factor is highly conserved within Lepidoptera. While in *Drosophila melanogaster*, the Sxl gene product is the key sex-determination factor, being required for female germline development, in the silkmoth *B. mori* it is required for male fertility (19). Constructing a targeted *sxl* insertion mutant that resulted in disruption of an RNA recognition motif (RRM) in both of *Bombyx*’s two *sxl* transcripts caused abnormal spermatogenesis in the testis, affecting apyrene but not eupyrene sperm. Nevertheless, the affected insects were male sterile; although eupyrene sperm were transferred apparently normally to the spermatophore during mating by the mutant Δ*sxl* males, no apyrene sperm could be detected there, and in their absence the eupyrene sperm were unable to migrate to the spermatheca. The fertility of the female could be rescued by a second mating with an infertile triploid male (which transfers apyrene sperm normally), indicating with considerable certainty that it is the lack of normal apyrene sperm that is responsible for the infertility of Δ*sxl* males.

The introduction in 2012 of the versatile and efficient CRISPR-Cas9 (Clustered Regularly Interspaced Short Palindromic Repeats/Cas9) technique for gene editing of eukaryotic genomes (157) effectively made the less tractable ZNF and TALENS systems redundant. The CRISPR/Cas9 system is derived from previous extensive study of the co-evolutionary interactions of bacteria and the viruses that attack them (158). Its potential for editing lepidopteran pest genomes was quickly grasped, when it was used to knock out a color-marker gene in *B. mori* (159).

Several of the gene editing projects that have been conducted in *Bombyx* and other Lepidoptera with CRISPR/Cas9 have targeted genes that are expressed in the testis and have been hypothesized to be involved in either sex determination or spermatogenesis, usually on the basis that their *Drosophila* homologues are known to affect fertility and/or sperm production (see **Table 3**). Almost all these papers describe studies clearly aimed at creating male-sterile insects suitable for SIT or similar control techniques.

**Table 3:** Targeting sperm-related genes using TALEN and CRISPR/Cas9.

Species	Order, Family	Method	Mode of delivery	Targeted Gene	Gene Product	Function	Treatment effect	Effect on fertility	Reference
*Bombyx mori*	Lepidoptera, Bombycidae	CRISPR-Cas9	Egg injection	*igflp*	IGFLP	Insulin-like growth factor-like peptide	No effect on testis size, but ovary size is reduced	Female (F) fertility reduced but no effect on male (M) fertility	(59)
*Bombyx mori*	Lepidoptera, Bombycidae	CRISPR-Cas9	Egg injection	*doublesex*	Doublesex	Transcription factor, sex determining regulator	Malformed M and F genitalia	M and F infertility associated with disruption of M- and F-specific transcripts	(132)
*Plutella xylostella*	Lepidoptera, Plutellidae	CRISPR-Cas9	Egg injection	*doublesex*	Doublesex	Transcription factor, sex determining regulator	Malformed M and F genitalia	M and F infertility associated with disruption of M- and F-specific transcripts	(160)
*Ostrinia furnacalis*	Lepidoptera, Crambidae	CRISPR-Cas9	Egg injection	*doublesex*	Doublesex	Transcription factor, sex determining regulator	Malformed M and F genitalia	M and F infertility associated with disruption of M- and F-specific *dsx* transcripts	(161)
*Ostrinia furnacalis*	Lepidoptera, Crambidae	CRISPR-Cas9	Egg injection	*masculinizer*	Masculinizer	Regulates sex-specific splicing of dsx	Male embryonic lethality; malformed M (but not F) genitalia	M infertility associated with disruption of M- and F-specific *dsx* transcripts	(161)
*Agrotis ipsilon*	Lepidoptera, Noctuidae	CRISPR-Cas9	Egg injection	*masculinizer*	Masculinizer	Regulates sex-specific splicing of dsx	Male embryonic lethality; malformed M (but not F) genitalia	M infertility associated with disruption of M- and F-specific *dsx* transcripts	(162)
*Bombyx mori*	Lepidoptera, Bombycidae	CRISPR-Cas9	Egg injection	*maelstrom*	Maelstrom	RNA-binding protein, essential for spermatogenesis in *Drosophila*, participates in piRNA pathway	Arrests spermatogenesis in elongation stage, both eupyrene and apyrene sperm affected	M infertility, no effect on F fertility	(163)
*Bombyx mori*	Lepidoptera, Bombycidae	CRISPR-Cas9	Egg injection	*prmt5* and *vasa*	Prmt5 and Vasa	Prmt5 is a type II arginine methyltransferase, Vasa is a helicase	Disruption of either Prmt5 or Vasa causes infertility	both M and F infertile	(164)
*Bombyx mori*	Lepidoptera, Bombycidae	TALEN	Egg injection	*sex-lethal*	Sex-lethal	Transcription factor	Fewer apyrene sperm, eupyrene sperm normal but do not migrate from spermatophore to spermatheca	M fertility reduced	(19)
*Bombyx mori*	Lepidoptera, Bombycidae	CRISPR-Cas9	Egg injection	*sex-lethal*	Sex-lethal	Transcription factor, sex determining regulator	Fewer apyrene sperm with defective mitochondrial derivative, eupyrene sperm normal but do not migrate from spermatophore to spermatheca	M fertility reduced	(18)
*Spodoptera litura*	Lepidoptera, Noctuidae	CRISPR-Cas9	Egg injection	*sex-lethal*	Sex-lethal	Transcription factor, sex determining regulator	Some sperm fail to undergo spermiogenesis in testis, fewer mature sperm of both kinds	M fertility reduced	(165)
*Bombyx mori*	Lepidoptera, Bombycidae	CRISPR-Cas9	Egg injection	*poly(A)-specific ribonuclease-like domain-containing 1 (pnldc1)*	Pnldc1	PIWI protein	Eupyrene spermatogenesis is abnormal, release of eupyrene sperm bundles from testis into UVD is prevented	M infertility	(18)
*Bombyx mori*	Lepidoptera, Bombycidae	CRISPR-Cas9	Egg injection	*polyamine modulated factor 1 binding protein 1 (pmfbp1)*	PMFBP1	Function of protein uncertain	Normal apyrene sperm bundles; abnormal eupyrene sperm bundles, with nuclei mislocated and disordered; release of eupyrene sperm bundles from testes into UVD is blocked, no eupyrene sperm in spermatophore	M infertility	(166)
*Bombyx mori*	Lepidoptera, Bombycidae	CRISPR-Cas9	Egg injection	*hua enhancer 1 (hen1)*	Hen1	Methyltransferase that modifies piRNAs	Aberrant spermatogenesis only in eupyrene cysts	Both M and F Hen1 mutants are infertile	(167)
*Bombyx mori*	Lepidoptera, Bombycidae	CRISPR-Cas9	egg injection	*siwi and ago3*	Siwi and Ago3	PIWI proteins	No effect on male (or female) fertility, suggesting that these components of PIWI pathway are not essential for spermatogenesis	No effect	(167)
*Bombyx mori and Plutella xylostella*	Lepidoptera, Bombycidae and Plutellidae	CRISPR-Cas9	egg injection	*serine protease 2*	Serine protease 2	Initiatorin-like peptidase, activates both apyrene and eupyrene sperm in spermatophore	Both apyrene and eupyrene sperm appear normal and appear in the spermatophore but fail to fertilise eggs	Dominant inheritable M infertility	(108)
*Spodoptera litura*	Lepidoptera, Noctuidae	CRISPR-Cas9	egg injection	*serine protease 2*	Serine protease 2	Initiatorin-like peptidase, activates both apyrene and eupyrene sperm	Both apyrene and eupyrene sperm appear normal and appear in the spermatophore but fail to fertilise eggs	Dominant inheritable M infertility	(168)
*Bombyx mori*	Lepidoptera, Bombycidae	CRISPR-Cas9	egg injection	*serine protease 1*	Serine protease 1	seminal fluid protein; proteinase of previously unknown function	Failure of eupyrene sperm bundle dissociation	Dominant inheritable M infertility	(169)
*Bombyx mori*	Lepidoptera, Bombycidae	CRISPR-Cas9	egg injection	*sfp62*	SFP62	seminal fluid protein, expressed in testis and in SV	Immotile apyrene sperm in spermatophore	Dominant inheritable M infertility	(170)
*Spodoptera frugiperda*	Lepidoptera, Noctuidae	CRISPR-Cas9	egg injection	*obp27*	OBP27	Odorant binding protein, sperm chemotaxis?	Fewer pairings, longer duration of mating	M fertility reduced	(171)

Many used gene editing to disrupt the lepidopteran sex-determination pathway, a popular target being the conserved autosomal sex-determining gene *doublesex* (*dsx*). This gene encodes a transcription factor that regulates a large number of downstream genes, specifying either male or female development according to its alternative splicing (130).

Most is known about the role of *dsx* in lepidopteran sex determination for the silkmoth *B. mori*. In brief, female development in *Bombyx* results from the presence on the single female-determining W chromosome of a region of DNA encoding multiple copies of the PIWI-interacting small RNA (piRNA) *feminizer* (*fem*) (172). Mature *fem* piRNA associates with the proteins SIWI and GTSF1 to form a silencing complex that silences the female insect’s *masculinizer*, a male specific gene found on the Z chromosome that encodes a CCCH-tandem zinc-finger protein found only in Lepidoptera. *fem* piRNAs are fully (*Bombyx*) or partially (*Plutella*) complementary with the sequence of *masc* mRNA, and it has been hypothesized that the *fem* piRNAs of these two species have evolved through convergence (173). In the absence of Masc protein, a female-specific transcript (DSXF) comprising exons 1-4 of the *dsx* gene is produced, which directs female development of the insect. In ZZ male moths, which lack a W chromosome, *fem* is absent, so that *masc* can be expressed. Masc in turn regulates mRNA splicing of *dsx* so as to produce a male-specific isoform (exons 1,2 and 5), which directs formation of the protein Dsxm and results in male development. Masc is also required for dosage compensation of genes located on the Z chromosome (172).

Disruption of *dsx* by gene editing in a number of lepidopteran species (see **Table 3**) effectively produces either completely or partially feminized adult moths with loss of male fertility, but since dsx mutations also result in abnormal genitalia and sexual behavior, these insects would not necessarily be useful in SIT programs. Moreover, it is not clear that *dsx* would be a suitable target in other lepidopteran species, in which much less is known about sex determination. It is certain that the situation is different in those species, e.g. bagworms (Psychidae) that lack a W chromosome and instead use a Z0/ZZ sex-determining system (174). There are also other species, such as the wild silkmoth *Samia cynthia*, which have a W chromosome but do not use it in sex determination (175). The role of female-specific piRNAs in regulating *masc* in the Asian corn-borer, *Ostrinia furnacalis* evidently differs from what happens in *B. mori* (176, 177). Despite such uncertainties about the upstream control of *dsx*, the role of sex-specific splicing of *dsx* transcripts in sex determination is generally agreed to be at least widespread among Lepidoptera.

Disruption by gene editing of several other genes, however, has specific adverse effects on spermatogenesis, disrupting the production of both eupyrene and apyrene sperm. They include *maelstrom* (163) as well as *prmt5 and vasa* (164). All three of these genes are known to be involved in the Piwi system, by which the germline is protected against the disabling consequences of transposon mobilization, and which is also required for sex determination in *Bombyx*. Some components of the Piwi system are however specialized to particular functions, and disruption of two other known Piwi genes, *siwi* and *ago3*, did not affect either male or female fertility (167).

Interestingly from the point of view of the present paper, however, mutating another gene, *hen1*, was shown specifically to disrupt formation of only eupyrene sperm, while still another gene, *sex-lethal*, affects only apyrene sperm. It is remarkable that targeting either of the sperm morphs while leaving the other unharmed is in each case sufficient to render the male moth sterile. That this is due solely to functions performed by the affected sperm type itself is shown by the fact that in each case, fertility can be rescued by allowing the female an additional mating with a different male possessing the other type of sperm. Thus, a second mating with a sterile triploid strain (which lacks eupyrene sperm but has normal apyrene sperm) is sufficient to restore fertility to *sex-lethal* mutant males that have normal eupyrene sperm but which lack apyrene sperm (19), while double-mating with apyrene-deficient *sex-lethal* mutants rescues the *ΔPMFBP1* infertility phenotype (166). These results provide direct experimental confirmation of the hypothesis that the function of apyrene sperm is solely to ‘help’ eupyrene sperm.

## 9 Male reproduction as a target for lepidopteran pest control

### 9.1 RNA interference for lepidopteran pest control

Interference with insect reproduction has long been considered to have excellent potential for targeted pest control (12). In particular, it offers promise in preventing the build-up of damaging pest numbers in the case of migrant pests; many of the most damaging lepidopteran pest species are migrants (178) and targeted methods of preventing their reproduction once arrived in new location could be combined with monitoring and forecasting services. Although there is still plenty of opportunity to learn more, we now know enough about genes that control the development and functioning of male reproduction in Lepidoptera to make serious efforts to interfere specifically with them; this involves not only sex-determination, but also both varieties of spermatogenesis, as well as activation of sperm on transfer to the female. One or more of these genes could in principle be targeted in practical pest control to cause male sterility. This could be achieved by using RNAi as mediated by dsRNAs, siRNAs or miRNAs to silence the genes in question.

RNAi-based pest control has much to recommend it; especially when combined with conservation biocontrol it offers the prospect of greatly reduced environmental impact (179). RNAi is highly specific so that the likelihood of off-target effects on beneficial insects or other organisms is reduced. It would have the important advantage of exerting immediate effects on the treated generation of pest insects, so that there would be little delay in suppressing population growth. Moreover, once effective methods to deliver dsRNAs or miRNAs to pests have been perfected, it should be relatively easy to adapt them to new dsRNA cargos effective against new pests. This would be much more specific than using toxic chemical sprays. A snag is that the use of synthetic dsRNAs as sprays is likely to be much more expensive than most small-molecule chemical pesticides, although costs would probably decrease if the technology were widely adopted (180).

Unfortunately, however, lepidopteran pests are in general refractory to RNAi. **Table 2** lists a number of laboratory studies that have demonstrated the feasibility of this approach to modulate male reproduction. It is notable that the number of lepidopteran species that have been shown to be susceptible to reproductive control by RNAi is far fewer than the number of non-lepidopteran species. The relatively poor response of Lepidoptera to RNAi treatments compared to other insect orders is almost certainly the main reason for this lack of success.

A particular problem is that many insects, and especially Lepidoptera, produce dsRNases that destroy foreign dsRNAs (181); these enzymes are expressed both in the gut (182, 183) and in hemolymph (184–186). Many studies have shown that dsRNA given to pest Lepidoptera by the oral route can be effective in producing useful RNAi, but in other cases the presence of RNase activity in the gut has prevented knockdown of the targeted genes by feeding dsRNA (182). One possibility to overcome this limitation would be to use RNase-inhibiting chemical adjuvants in RNAi treatments, but this might have unpredictable environmental effects.

Intracellular delivery of the dsRNA cargo, as used to knock down serine protease 2 in *H. cunea* (137), has been shown to be effective in genetically engineered insects, thus overcoming the RNase-related problems mentioned above. This demonstrates that in principle an RNAi approach to the disruption of lepidopteran male reproduction could work, but it is hard to see how a strategy of this type could be used in practical pest control.

Another possibility to explain the poor response of Lepidoptera to RNAi is that many lepidopteran species are afflicted with chronic viral infections, which may adversely affect the host’s RNAi response through viral RNAi inhibitor genes (187). Still another issue is that in *M. sexta*, two essential proteins of the lepidopteran RNAi cellular machinery, the proteins Argonaute-2 (Ago-2) and Dicer-2, are expressed only transiently in response to the detection of dsRNA (188) and this may also be the case in other lepidopterans. Lack of Dicer-2 would adversely affect production of siRNAs from dsRNA, while lack of Ago-2 would reduce the efficiency of siRNA-mediated slicing. It has been shown (189) that overexpressing Ago-2 in *B. mori* improves the efficiency of RNAi in that insect. Co-administering a dsRNA-like chemical adjuvant might be able to induce synthesis by the pest of the missing RNAi cellular machinery, but this would also pose the risk of unexpected non-target effects.

Although spraying dsRNA on crop plants has been shown to be effective as a practical method of adversely affecting the fitness of several insect pests, its utility in practice requires that steps are taken to ensure the persistence of the dsRNA on the plant surface for a sufficient length of time, as well as its efficient uptake by the pest. Coating dsRNA onto various kinds of cationic nanoparticles, encapsulation within liposomes, as well as expressing dsRNAs within microorganisms are among the approaches that have been tried (190–193). An alternative would be to use chemically modified dsRNAs that are less susceptible to nuclease action (194).

Genetic engineering of crop plants to express dsRNAs (sometimes termed Host-Induced Gene Silencing – HIGS) at least potentially offers a methodology to overcome most of the above problems. Not only is the task of synthesizing the active ingredient of the pest-control method subcontracted to the crop plant itself, but due to its location within the plant, the dsRNA is to at least some extent protected from environmental degradation. An approach to improve the RNAi response in the pest that appears to hold particular promise is to specifically engineer the host plant to simultaneously express multiple RNAi-inducing siRNAs directed against several different cellular targets (195, 196).

Expressing dsRNA sequences within chloroplasts of host plant cells results in a much higher yield of dsRNA than is obtained when the same dsRNA is expressed in cytoplasm (Zhang et al., 2015). This seems a promising method of dsRNA delivery to herbivorous insect pests and has led to the development of an effective method of delivering a dsRNA directed against β-actin, thus protecting potato plants against the Colorado potato beetle *Leptinotarsa decemlineata* (197). Unfortunately, recent work (Fu et al., 2020) has shown that this methodology is not universally effective. Transplastomic delivery of dsRNA in tobacco plants proved to be ineffective against *H. armigera* larvae; it was suggested that this is because in contrast to the case where dsRNAs are expressed in cytoplasm, plastid-expressed dsRNAs are much less efficiently processed to siRNAs. It appears that in this insect full length dsRNAs are rapidly degraded in the insect’s gut, whereas the siRNAs derived from them are not. As noted above, in at least some lepidopterans the enzymatic machinery for producing siRNAs is not continually present, so that it may be advantageous to cause the plant to supply siRNAs rather than dsRNA. On the other hand, it was also found in other work that when artificial microRNAs (amiRNAs) were expressed in plant cells, although these were less efficiently processed to siRNAs in the plants (*Nicotiana benthami*) than in the insects (*H. armigera*) that ate them (196), the effectiveness of the resultant RNAi in the insect was actually enhanced. Given the potential for a wide range of variation in the processing of dsRNA or hp-RNA into siRNAs between different genes, host plants and pest insects, it is at least evident that efficient delivery of RNAi-inducing agents through plant genetic engineering is more complex than was first envisaged.

While much has been achieved in developing practical RNAi insect control schemes, mostly directed against other non-reproductive targets (198–201), the technique has yet to be deployed on a large scale, and many operational difficulties remain (192, 202). Limitations of these kinds may explain while RNAi-based crop protection in general, especially that based on plant genetic engineering, looks promising, its utility against Lepidoptera has not yet proved to be the success that was initially anticipated (203). It is beyond the scope of this paper to consider in detail the pros and cons of various RNAi-based crop protection strategies, which have been well reviewed elsewhere e.g (180).

### 9.2 Gene editing for SIT

In contrast with RNAi-based approaches, the generation of targeted mutants using gene editing techniques is in principle less susceptible to failure based on the exact deployment method. Targeted mutation of the chosen gene is done in the laboratory and can be verified before proceeding to practical pest control. So far, gene editing has been used mostly to test hypotheses about gene function (see above). But gene editing could in principle also be used in practical insect pest control as a means of introducing fertility-limiting genes into the pest population through the sterile insect technique (SIT) (204, 205).

Classical SIT (13) generates dominant lethal mutations in laboratory-bred insects and then mass releases the resulting insects into wild populations. Originally viewed as a way to eradicate damaging pests, SIT is now more often seen as a method for suppressing targeted populations to levels below economic thresholds (206). It has been successful against a number of insect pests, especially dipteran vectors of disease and crop pests, and can minimize the environmental damage associated with pest control by reducing chemical pesticide use (207, 208). Ionizing radiation is most commonly used in mutagenesis, but chemosterilants can also be used. Once released, the radiation-damaged insects mate with wild conspecifics but are effectively sterile because their offspring fail to hatch. With a sufficiently high ratio of irradiated insects to wild ones, this suppresses the growth of the pest population. Sterile males contribute most to population suppression because they can mate with more than one wild female. It is important to realize that this approach need not (and usually does not) involve any effect at all on the production of sperm by the sterilized male insects.

There are practical limitations to the classical SIT approach, however. A particular disadvantage is the depressed vigor typically associated with radiation- or chemosterilant-induced sterility. This limits the ability of treated males to compete with wild males for mates, and is a consequence of the random nature of the DNA damage (double stranded breaks and transpositions) typically caused by radiation and chemosterilants (40). The problem is particularly severe in Lepidoptera, which due to the holokinetic nature of their chromosomes are exceptionally resistant to radiation, so that high doses of radiation must be used to sterilize them (40). This limitation can be overcome to some extent by exposing insects to lower, substerilizing doses of radiation. Following adult emergence, the irradiated moths are allowed to reproduce to produce (unirradiated) progeny, which mate readily with wild insects but in doing so exhibit a higher degree of sterility than the originally-irradiated parental insects. Moreover, the F1 generation is markedly male-biased, making it immediately useful for release in SIT programs (209–211). This is usually termed ‘inherited sterility’ or F1 sterility. There have been a number of notable cases in which SIT based on inherited sterility of this type has achieved area-wide success in controlling lepidopteran insects; notably, Pink Bollworm (*Pectinophora gossypiella*), a serious pest of cotton, has been eradicated from Southern California, USA, and Northern Mexico using a combination of SIT and BT cotton (40). Nevertheless, the SIT technique has not achieved mainstream status against lepidopteran pests.

A problem that limits area-wide deployment of classical radiation- or chemosterilant-based SIT is that it is costly, requiring central coordination and capital expenditure on radiation sources as well as intensive labor in insect rearing. Further, its efficiency is limited unless only male insects are released, and sorting according to sex is slow and expensive. The efficiency of SIT could be considerably increased if males could be sterilized and females eliminated by genetic means (212, 213). Although balanced-lethal genetic sexing strains have been developed for the domestic silkmoth, *B. mori* and the Flour Moth, *Ephestia kühniella*, they are challenging to maintain, requiring regular checking to detect genetic breakdown. Solutions of this type have been described as “impractical” for pest control because they are too labor-intensive (40).

Genetic manipulation strategies to solve these limitations of SIT are termed ‘Genetic Pest Management’ (GPM) (214). As described in section 9, gene editing using CRISPR/Cas9 has already proved of great value in experimental investigations of gene function in insects and is an obvious choice to use in constructing genetic sexing lines for SIT in Lepidoptera. Reflecting the considerable interest in using the CRISPR-Cas9 technique in pest control (205), several recent publications have reviewed its application to lepidopteran pests (204, 215–217). **Table 3** lists several examples.

One ‘first generation’ approach to GPM depends on the same general principles as SIT, but instead of using radiation to induce mutations randomly in the genome uses genetic engineering to introduce a lethal transgene. This is female-specific Release of Insects carrying a Dominant Lethal mutation (fsRIDL). Genetically modified fsRIDL lines of Diamondback Moth, *Plutella xylostella* and *Pectinophora gossypiella* have been created (218, 219). They possess a conditionally-expressed, female-specific dominant lethal gene, encoding the tTA transactivator of the ‘tet-off’ system, which is fatally overexpressed exclusively under the control of a female-specific transcript of the sex-determining gene *doublesex*. Both sexes can be maintained in continuous culture by rearing under conditions that prevent expression of the mutant gene product in the susceptible sex (continual presence of the antibiotic chlortetracycline), but when antibiotic is omitted from larval food, the female insects die, leaving all-male cultures that can transmit the dominant lethal gene to offspring but do not express it themselves. When these males are released to mate with wild congeners, an F1 generation is produced that is highly biased towards males, thus continuing the SIT strategy. The mating competitiveness of males from the engineered line of *P. xylostella* was, however, significantly less than that of the wild type (218), presumably because the tet-off system imposes significant fitness costs on the transgenic insects that reduce their ability to compete as mates.

Costs of this kind can be avoided by using gene editing to produce ‘precision guided’ SIT (pgSIT) systems that disrupt only chosen genetic loci to generate insects that retain full sexual vigor. This has been hailed as ‘second-generation GPM’ (214). pgSIT has been demonstrated to be achievable for *Drosophila* (220) and *Anopheles* (221). In brief, separate homozygous lines were engineered, in one of which Cas9 was expressed under the influence of a strong promoter, while in other lines double guide RNAs (dgRNAs) were expressed, which were complementary to genes required for full sexual competence. In the original work on *Drosophila* (220), the presence of the *cas9* and dgRNA transgenes in a single genome was lethal in females and induced sterility in males. Target genes were selected from various female specific forms of *sxl*, and *dsx*, as well as male-specific genes that are expressed only in the testis, such as *β-tubulin*, *fuzzy onions*, *protamine A* and *spermatocyte arrest*. Complementary sequences specific to these genes were engineered into the genomes of single guide (*sg*) lines, which were confirmed to have the expected properties before being combined through standard genetic techniques into a number of homozygous lines that combined various pairs of female-specific and male-specific guide sequences. When the *dg* lines were crossed with the *cas9* line, the resulting female offspring were flightless and were thus easily removed leaving the remaining insects as 100% males, which although completely sterile competed equally with wild type males and survived significantly longer than them. This is an impressive achievement, but it still does not completely eliminate the need to maintain multiple insect lines and to sort them according to sex. Subsequently, however, the same research group has demonstrated the utility of a method of temperature-inducible control of Cas9 expression that completely eliminates the need to generate a cross between two separately maintained insect lines (222).

There is no reason to think that similar self-sexing, targeted sterile but fully vigorous lines suitable for practical pgSIT cannot be created with lepidopteran insects, although a successful outcome has not yet been reported. Although Lepidoptera are unusual among insects in their female heterogametic chromosomal sex determination, this should not affect the outcome of such experiments. As is the case in *Drosophila*, the genetic sex-determination method of Lepidoptera is itself a potential target for gene editing a self-sexing SIT line. Although the lepidopteran sex determination cascade is still incompletely understood, enough is known about the process in *B. mori* (223), and *P. xylostella* (173), to test the hypothesis that its disruption could be the basis of potential pest control methods. Nevertheless, we suggest here that it is inevitable that disruption of *dsx*-mediated sex determination is always likely to produce sterile males that do not compete well with wild insects. We predict that it will be better to target downstream male-specific genes that leave sexual anatomy and behavior unaffected, but which interfere with the production of fertile sperm. Since apyrene sperm are in the majority in most Lepidoptera and are known in at least some cases to influence female post-mating behavior, while only eupyrene sperm can fertilize eggs, we suggest that interference with eupyrene sperm formation is a particularly attractive target.

### 9.3 Homing gene drives

Much interest is currently focused on the use of gene drives for insect control, in which a lethal (or at least detrimental) mutation or transgene is preferentially inherited (i.e. it is a selfish genetic element, passed on at each generation at a frequency greater than that expected from Mendelian theory). Once such a gene drive is introduced into a pest population, the detrimental gene(s) will inevitably spread, its effects on fitness causing the population size to diminish (224). Such selfish elements exist in nature, but recent advances in gene editing techniques such as CRISPR/Cas9 have made it relatively easy to create synthetic gene drives. Most interest is attracted by homing endonuclease gene drives (HEG), in which the gene encoding a sequence-specific endonuclease such as Cas9 is inserted into a target DNA sequence that is necessary for fitness. Initially the HEG is present in the genome of germ cells in only one copy (i.e. the cell is heterozygous for the gene drive element). Under certain conditions, the HEG creates a double-stranded break in the corresponding DNA target (which does not yet contain the HEG) but does not attack the site that contains its own gene, because the target sequence is disrupted by the inserted transgene. During the process of repairing the break, the HEG-containing sequence acts as a template and is copied to the repair site, so that the cell becomes homozygous for the selfish element. Typically, the HEG acts as a recessive gene that is either directly lethal or imposes sterility when homozygous. In the case of sterility, just as in SIT, when a sufficient proportion of homozygous insects is present, population size will dwindle. But it is not necessary as in SIT to go on introducing more sterile insects into the population, because the gene that causes sterility continues to be inherited for an increasingly large proportion of heterozygotes in each generation. Depending on the parameters of preferential inheritance and loss of fitness, such gene drives are capable of driving the pest population extinct (224).

Such HEGs have the potential to be used in practical insect control (40, 214, 224–227). A successful demonstration of the potential to use the CRISPR-Cas9 gene editing system for HEG-based pest control used the mosquito *Anopheles gambiae* (225). A transgenic sequence encoding a guide sequence (gDNA) was inserted into the sex-specifying *dsx* gene, at precisely the locus that is required for female-specific splicing of *dsx*, but which does not interfere with male-specific splicing. In females, the resulting disruption of DsxF is recessive, but when two copies of the disrupted allele are inherited, its normal sexual development is prevented resulting in the development of an effectively sterile intersex adult. When mosquitoes bearing this gene drive were introduced into caged populations, the recipient cages quickly crashed and went extinct.

There are advantages in locating HEGs within genes that are subject to strong stabilizing selection. This is in part because the chosen target sequence should if possible be present in all members of the population, and partly because homing gene drives are in principle susceptible to the evolution of resistance through mutations in the target DNA sequence. Since mutations within highly conserved sequences are themselves likely to be disadvantageous, placing the HEG within one of these will minimize the probability of resistance arising there (214, 224).

Homing gene drives do not necessarily have to be based on sexually divergent traits (any fitness-conferring gene can be targeted) but interfering with sex is an obvious way to make the gene drive reliably deleterious to fitness. Most interest so far has concentrated on the direct disruption of the most conserved components of sex determination GRNs. As noted above, disruption of eupyrene spermatogenesis might be particularly useful.

Other genetic strategies using CRISPR-Cas9 to achieve similar ends are available. An example is the use in a genetic sexing line of *Anopheles* mosquitoes of the ‘X-shredder’ strategy. This uses guide sequences that complement repetitive genomic sequences that uses a different homing endonuclease, PpoI, originally derived from the slime mold *Physarum polycephalum*. This enzyme selectively targets repetitive sequences in ribosomal DNA, present in numerous copies on the X-chromosomes; when expressed under the control of a promoter used only in spermatocytes, it ‘shreds’ X-chromosomes in developing sperm during spermatogenesis (228). The outcome is that a high proportion of viable male gametes contain a Y-chromosome, ensuring the production of all-male, or nearly all-male progeny. By targeting numerous target sites, this strategy reduces the possibility of resistance developing through mutations in the target sequence. Another strategy to minimize resistance would be to produce ‘multiplex’ HEG lines that are targeted to different closely linked gene sequences (229).

In Lepidoptera, an analogous gene drive strategy would be to engineer a ‘W-shredding’ nuclease onto the Z chromosome (230). This would result in the death of female embryos, leaving a high proportion of F1 male offspring carrying the drive. Simulations show that this would be a very successful pest suppression strategy, as long as few daughters are produced is high and the probability of resistance mutations arising is low. Since the W chromosome consists largely of rapidly-evolving and highly repetitive DNA elements, and contains few if any genes (231), it is uncertain that such a strategy is feasible.

The theoretical and practical problems associated with creating efficient pesticidal gene drives, the ethical and conservation implications of introducing homing gene drives into the environment, and possible ways in which gene drives could be recalled, are beyond the scope of this paper, but have been extensively discussed (214, 232–236).

## Author contributions

RS, PY and SR prepared and revised draft versions, then prepared the final manuscript. All authors contributed to the article and approved the submitted version.
