# Corrigendum: Dichotomous sperm in Lepidopteran insects: a biorational target for pest management

**DOI:** 10.3389/finsc.2024.1343089

**Published:** 2024-02-16

**Authors:** Rakesh K. Seth, Priya Yadav, Stuart E. Reynolds

**Affiliations:** ^1^ Department of Zoology, University of Delhi, Delhi, India; ^2^ Department of Life Sciences, University of Bath, Bath, United Kingdom; ^3^ Milner Centre for Evolution, University of Bath, Bath, United Kingdom

**Keywords:** spermatozoa, sperm activation, sperm motility, initiatorin, serine endopeptidase, Lepidoptera, pest management, RNAi

In the published article, there was an error in **Figure 3**. At the right-hand side of the figure, the micrographs showing apyrene and eupyrene sperm bundles were inadvertently transposed. The original caption is correct. The corrected **Figure 3** and its caption appear below.

**Figure 3:**
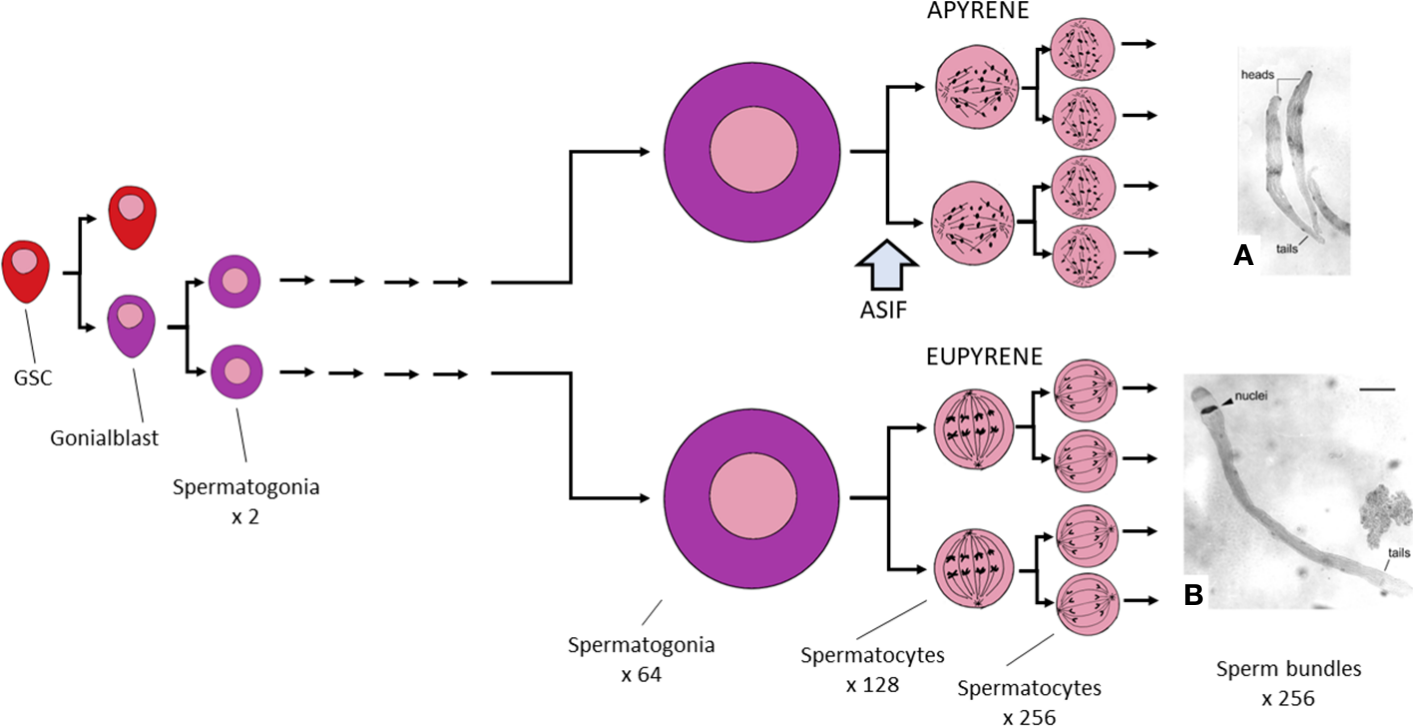
Graphic summary of Lepidopteran spermatogenesis. Germline stem cells (GSC) divide asymmetrically to produce a single self-renewing GSC and a single gonialblast. The latter divides to produce two spermatogonia, which then grow remarkably in size while also undertaking five rounds of mitosis. Development is indistinguishable in cysts that will eventually produce eupyrene and those that lead to apyrene sperm. Spermatogonia now enter the prophase of meiosis I, but arrest at this point until the block on meiosis is lifted, probably by exposure to ecdysteroid in the absence of juvenile hormone (JH). At this time, the spermatocyte will become committed to producing a eupyrene sperm unless it is also exposed to a (hypothetical) apyrene sperm stimulating factor (ASIF) that acts to commit the cell to producing an apyrene sperm (see the text for further discussion of this point). From this time onward, eupyrene and apyrene cysts differ in many ways. **(A)** shows two apyrene sperm bundles; **(B)** shows a single eupyrene sperm bundle, both from *Ephestia kühniella*; **(A, B)** are from reference (40), reproduced with permission.

The authors apologize for this error and state that this does not change the scientific conclusions of the article in any way. The original article has been updated.

